# Genetic Links Between Cancer and Coronary Atherosclerosis: A Mendelian Randomization Analysis

**DOI:** 10.1155/humu/5997499

**Published:** 2026-07-08

**Authors:** Yanan Fan, Miaomiao Liu, Lifei Zhang, Fei Liu, Jiantao Dong, Mengyang Zhang, Lixiao Zhang, Yun Sun, Wenjing Yao, Wei Geng

**Affiliations:** ^1^ Hebei General Hospital, Shijiazhuang, China, hebmu.edu.cn

**Keywords:** colorectal cancer, coronary atherosclerosis, hepatic cancer, lung cancer, mendelian randomization, ovarian cancer, pancreatic cancer

## Abstract

**Background:**

While observational studies have indicated a potential link between cancer and coronary atherosclerosis, the relationship remains obscured by confounding factors. Elucidating a shared genetic basis may uncover common pathophysiological pathways.

**Methods:**

We employed a two‐sample Mendelian randomization (MR) approach to evaluate causal relationships between 23 cancer types and coronary atherosclerosis. Reverse MR analysis was also conducted to explore potential bidirectional effects. Given the exploratory nature of this pan‐cancer analysis, we report nominal significance (*p* < 0.05) without multiple‐testing correction.

**Results:**

Forward MR analysis identified nominally significant associations between coronary atherosclerosis and five cancer types; however, after rigorous sensitivity analyses, only the inverse associations with pancreatic cancer (OR = 0.90, 95*%*CI = 0.83–0.98 and hepatic cancer (OR = 0.87, 95*%*CI = 0.81–0.94 remained robust. Reverse MR analysis suggested that genetic predisposition to ovarian cancer was associated with lower odds of coronary atherosclerosis(OR = 0.97, 95*%*CI = 0.94–0.99 ), though the effect size was modest.

**Interpretation:**

This comprehensive large‐scale MR study provides hypothesis‐generating evidence for a bidirectional genetic interplay between cardiovascular and oncological diseases. These findings point to a complex interplay between these conditions, paving the way for future investigations into shared mechanisms and integrated therapeutic strategies. However, all associations should be interpreted as suggestive rather than definitive causal evidence, and validation in independent cohorts is warranted.

## 1. Introduction

Cancer and coronary atherosclerosis are significant public health concerns, contributing to substantial morbidity and mortality worldwide [1, 2]. Observational studies have suggested potential associations between these two conditions [3]; however, the nature and direction of this relationship remain unclear due to confounding factors and reverse causation. Recent advancements in genomics have prompted researchers to explore the role of genetic factors in the interplay between these diseases. Mendelian randomization (MR) is a method that utilizes genetic variants as instrumental variables (IVs), effectively addressing the confounding and reverse causation issues prevalent in observational studies [4]. Previous studies have indicated complex interactions between certain types of cancer and cardiovascular diseases [5, 6]. For instance, breast and renal malignancies are closely associated with the presence and development of hypertension [7]. Chronic renal hypoxia in renal cell carcinoma has been shown to lead to increased lipid peroxidation and overexpression of angiotensin receptors, while downregulating angiotensin‐converting enzyme [8]. From the perspective of breast cancer, similar mechanisms have been proposed, including chronic tissue hypoxia leading to increased inflammation, which in turn promotes the formation of reactive oxygen species [9].

However, research specifically examining the genetic associations between cancer and coronary atherosclerosis remains limited. This study is aimed at filling this gap by employing MR to investigate causal relationships between 23 different types of cancer and coronary atherosclerosis. A review of existing literature reveals that cardiovascular disease risk factors are often linked to the incidence of specific cancer types. For example, smoking and obesity are recognized as common risk factors for both cardiovascular disease and certain cancers [10, 11]. Additionally, inflammatory responses may play a pivotal role in the pathogenesis of both diseases, offering a new perspective on their interrelationship [12, 13]. Nevertheless, most existing studies rely on observational data, lacking definitive causal validation. Therefore, utilizing MR to explore the genetic associations between cancer and coronary atherosclerosis will provide more robust evidence. Through this research, we aim to elucidate the complex interplay between cardiovascular diseases and cancer, ultimately offering new insights for integrated prevention and treatment strategies.

## 2. Methods

### 2.1. Cancer and Coronary Atherosclerosis Data

The genome‐wide association studies (GWAS) IDs for 23 different types of cancer are ebi‐a‐GCST90018808, ebi‐a‐GCST90018875, ebi‐a‐GCST90018921, ebi‐a‐GCST90018929, ebi‐a‐GCST90018841, ebi‐a‐GCST90018893, ebi‐a‐GCST90018849, ebi‐a‐GCST90018803, ebi‐a‐GCST90018858, ebi‐a‐GCST004744, ukb‐a‐60, ebi‐a‐GCST90018799, ieu‐b‐4874, ebi‐a‐GCST90018817, finn‐b‐CD2_HODGKIN_LYMPHOMA, finn‐b‐C3_DLBCL, finn‐b‐CD2_TNK_LYMPHOMA, finn‐b‐C3_GBM, ieu‐b‐4912, finn‐b‐C3_MESOTHELIOMA, ebi‐a‐GCST90018888, ebi‐a‐GCST90018905, and finn‐b‐C3_TESTIS. The login numbers for coronary atherosclerosis are finn‐b‐I9_CORATHER. For sex‐specific cancers (e.g., ovarian and testicular cancers), we utilized sex‐combined GWAS summary statistics for coronary atherosclerosis due to data availability constraints. This approach may introduce sex‐related confounding and statistical noise, and thus the findings for these cancers should be interpreted with particular caution.

### 2.2. MR Analysis

MR leverages genetic variants as IVs to estimate the causal effect of an exposure on an outcome, minimizing the impact of confounding factors and reverse causality. In this study, we employed the two‐sample MR approach, where genetic associations with the exposure and outcome were obtained from independent GWAS. The primary method for MR analysis was the inverse variance‐weighted (IVW) approach, which combines the ratio estimates of individual single nucleotide polymorphisms (SNPs) by weighting them inversely proportional to their variance. Genetic variants were extracted as IVs using the default parameters of the TwoSampleMR package. Specifically, SNPs strongly associated with the exposure at genome‐wide significance (*p* < 5 × 10^−8^) were selected. To ensure the independence of the selected IVs, linkage disequilibrium (LD) clumping was performed with a threshold of *r* ^2^ < 0.001 and a clumping window of 10,000 kb. The IVW method assumes that all genetic variants are valid instruments, with no horizontal pleiotropy, providing a consistent estimate of the causal effect when this assumption holds. To account for potential horizontal pleiotropy, where genetic variants affect the outcome through pathways other than the exposure, we employed the MR‐Egger regression method. MR‐Egger allows for pleiotropy by including an intercept term, where a nonzero intercept suggests the presence of pleiotropy. The slope of the MR‐Egger regression provides an adjusted estimate of the causal effect, accounting for this bias. Heterogeneity across SNP‐specific causal estimates, which may indicate variability in the causal effect or the presence of invalid instruments, was assessed using Cochran′s Q‐test. A significant Q‐statistic (*p* value < 0.05) indicates the presence of heterogeneity, suggesting that the genetic variants may not all estimate the same causal effect or that pleiotropy may be present. To further validate the robustness of our results, we conducted a leave‐one‐out (LOO) sensitivity analysis. This method systematically excludes each SNP from the analysis one at a time, recalculating the overall causal estimate to identify any single SNP driving the results.

### 2.3. Statistical Analyses

All statistical analyses were conducted using R software (Version 4.3.1), with MR analyses performed using the TwoSampleMR package and additional bioinformatics analyses using relevant computational tools.

## 3. Results

**Figure 1:**
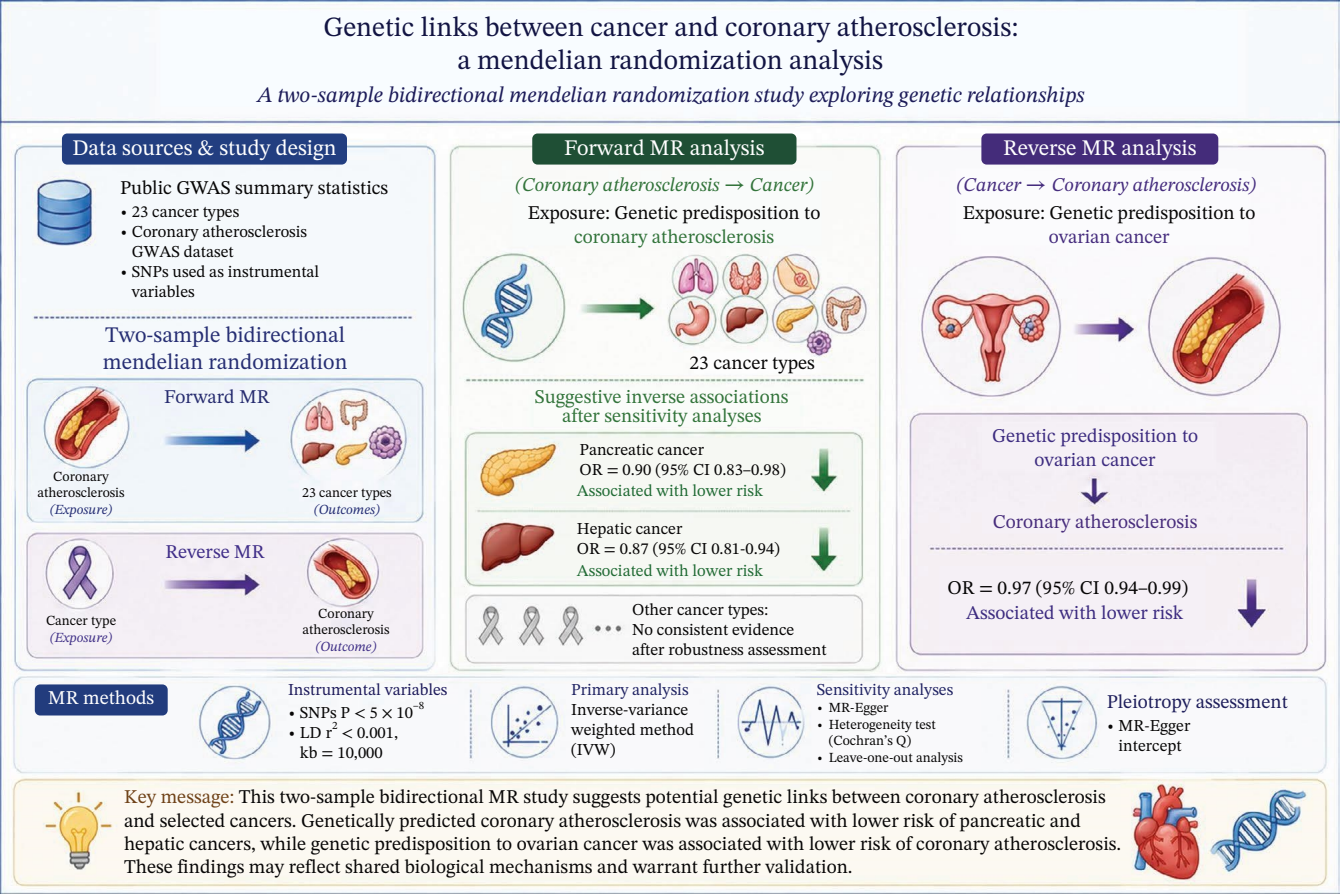
Study overview. Schematic of the bidirectional Mendelian randomization framework, illustrating the use of SNPs as instruments to assess the causal relationship between coronary atherosclerosis and multiple cancer types.

To provide an overview of the study design and major findings, a comprehensive graphical abstract was generated and is presented in Figure 1. This schematic summarizes the overall MR framework, including the selection of SNPs as IVs, the assessment of coronary atherosclerosis as the exposure, the evaluation of multiple cancer types as outcomes, and the bidirectional analyses conducted to explore potential reverse causality.

### 3.1. The Discussion of the Causal Effect Between Coronary Atherosclerosis and Cancer

**Figure 2:**
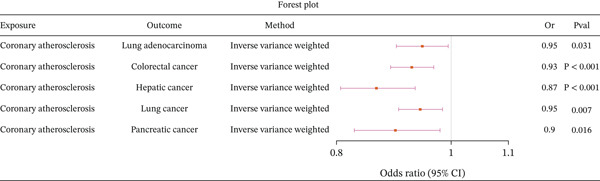
Analysis of the causal relationship between coronary atherosclerosis and various cancers using the IVW method.

We evaluated the causal relationships between coronary atherosclerosis and 23 different types of cancer, primarily utilizing the IVW method (Table S1). In the initial IVW analysis, we observed nominally significant associations (*p* < 0.05) between coronary atherosclerosis and five cancer types: lung adenocarcinoma, colorectal cancer, hepatic cancer, lung cancer, and pancreatic cancer. However, given the exploratory nature of this pan‐cancer analysis and the absence of multiple‐testing correction, these initial findings required further validation through sensitivity analyses. (Figure 2).

**Figure 3:**
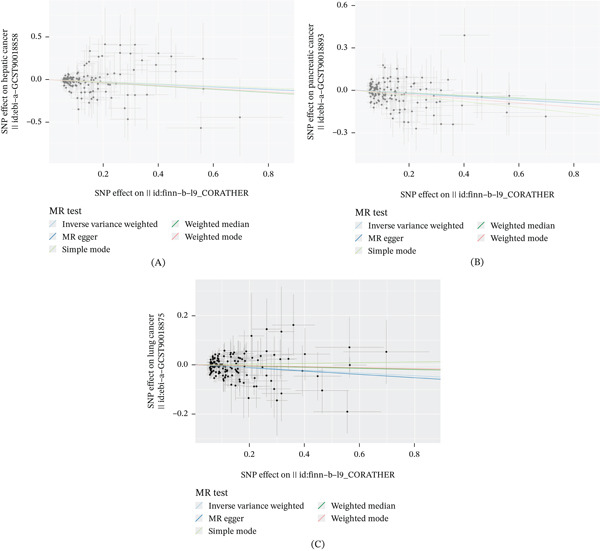
(A, B, C) Scatter plots demonstrating the robustness of results analyzing multiple causal relationships using various methods.

To verify the robustness of our findings, we conducted horizontal pleiotropy tests, heterogeneity tests, and LOO analyses. Horizontal pleiotropy was assessed using the MR‐Egger method, and the *p* values for the intercepts in MR‐Egger regression were all greater than 0.05, indicating no evidence of horizontal pleiotropy **(**Table 1 **)**. In the heterogeneity test, we found that the *p* values for the associations between coronary atherosclerosis and lung adenocarcinoma, as well as between coronary atherosclerosis and colorectal cancer, were less than 0.05, suggesting the presence of heterogeneity **(**Table 2 **)**. The LOO analysis demonstrated a consistent trend across all included SNPs (Figures S1, S2, S3), and scatter plots further confirmed the robustness of our findings (Figure 3A,B). However, according to the scatter plot results, we observed that the causal relationship between coronary atherosclerosis and lung cancer as determined by the weighted median method did not align directionally with the results from the other four methods (Figure 3C). Overall, our findings suggest a causal relationship between coronary atherosclerosis and pancreatic cancer (odds ratio [OR] = 0.90, 95% confidence interval [CI] = 0.83–0.98, *p* value < 0.05) as well as between coronary atherosclerosis and hepatic cancer (OR = 0.87, 95*%*CI = 0.81–0.94, *p* value < 0.05) (Figure 2).

**Table 1:** Horizontal pleiotropy assessment of coronary atherosclerosis and multiple cancers.

	id.exposure	id.outcome	outcome	exposure	egger_intercept	se	*p*
1	finn‐b‐I9_CORATHER	ebi‐a‐GCST004744	Lung adenocarcinoma || id:ebi‐a‐GCST004744	|| id:finn‐b‐I9_CORATHER	0.0013	0.005305	0.806899
2	finn‐b‐I9_CORATHER	ebi‐a‐GCST90018808	Colorectal cancer || id:ebi‐a‐GCST90018808	|| id:finn‐b‐I9_CORATHER	−0.00682	0.004301	0.115213
3	finn‐b‐I9_CORATHER	ebi‐a‐GCST90018858	Hepatic cancer || id:ebi‐a‐GCST90018858	|| id:finn‐b‐I9_CORATHER	0.00089	0.007874	0.910186
4	finn‐b‐I9_CORATHER	ebi‐a‐GCST90018875	Lung cancer || id:ebi‐a‐GCST90018875	|| id:finn‐b‐I9_CORATHER	0.001516	0.004356	0.728435
5	finn‐b‐I9_CORATHER	ebi‐a‐GCST90018893	Pancreatic cancer || id:ebi‐a‐GCST90018893	|| id:finn‐b‐I9_CORATHER	0.001889	0.008985	0.833846

**Table 2:** Heterogeneity assessment of coronary atherosclerosis and multiple cancers.

	id.exposure	id.outcome	Outcome	Exposure	Method	Q	Q_df	Q_pval
1	finn‐b‐I9_CORATHER	ebi‐a‐GCST004744	Lung adenocarcinoma || id:ebi‐a‐GCST004744	|| id:finn‐b‐I9_CORATHER	MR Egger	173.7306	123	0.001781
2	finn‐b‐I9_CORATHER	ebi‐a‐GCST004744	Lung adenocarcinoma || id:ebi‐a‐GCST004744	|| id:finn‐b‐I9_CORATHER	Inverse variance weighted	173.8154	124	0.002127
3	finn‐b‐I9_CORATHER	ebi‐a‐GCST90018808	Colorectal cancer || id:ebi‐a‐GCST90018808	|| id:finn‐b‐I9_CORATHER	MR Egger	240.3496	133	3.50E‐08
4	finn‐b‐I9_CORATHER	ebi‐a‐GCST90018808	Colorectal cancer || id:ebi‐a‐GCST90018808	|| id:finn‐b‐I9_CORATHER	Inverse variance weighted	244.8927	134	1.65E‐08
5	finn‐b‐I9_CORATHER	ebi‐a‐GCST90018858	Hepatic cancer || id:ebi‐a‐GCST90018858	|| id:finn‐b‐I9_CORATHER	MR Egger	116.1361	133	0.850807
6	finn‐b‐I9_CORATHER	ebi‐a‐GCST90018858	Hepatic cancer || id:ebi‐a‐GCST90018858	|| id:finn‐b‐I9_CORATHER	Inverse variance weighted	116.1489	134	0.864724
7	finn‐b‐I9_CORATHER	ebi‐a‐GCST90018875	Lung cancer || id:ebi‐a‐GCST90018875	|| id:finn‐b‐I9_CORATHER	MR Egger	142.1383	133	0.278057
8	finn‐b‐I9_CORATHER	ebi‐a‐GCST90018875	Lung cancer || id:ebi‐a‐GCST90018875	|| id:finn‐b‐I9_CORATHER	Inverse variance weighted	142.2676	134	0.296142
9	finn‐b‐I9_CORATHER	ebi‐a‐GCST90018893	Pancreatic cancer || id:ebi‐a‐GCST90018893	|| id:finn‐b‐I9_CORATHER	MR Egger	130.5626	133	0.543565
10	finn‐b‐I9_CORATHER	ebi‐a‐GCST90018893	Pancreatic cancer || id:ebi‐a‐GCST90018893	|| id:finn‐b‐I9_CORATHER	Inverse variance weighted	130.6067	134	0.566798

### 3.2. Reverse MR Analysis

**Figure 4:**
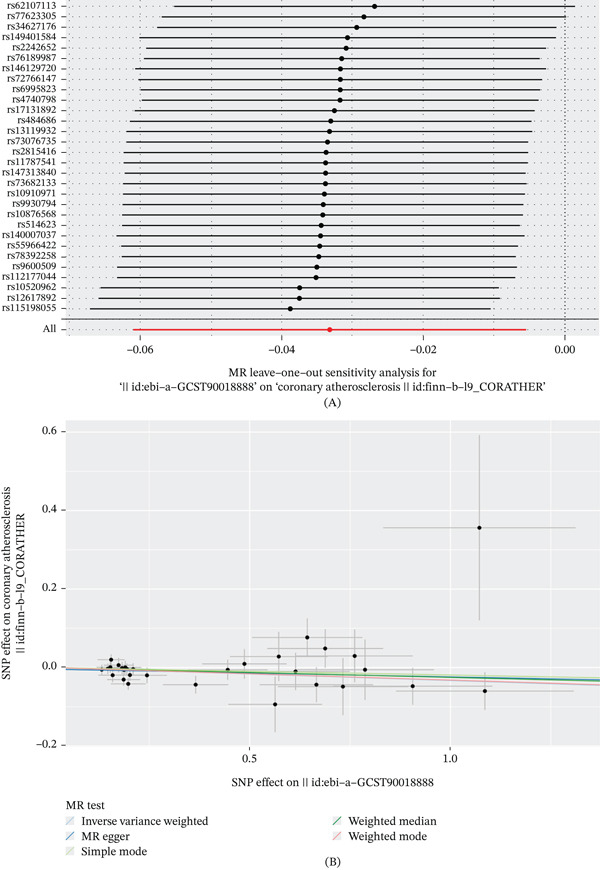
(A) Mendelian randomization leave‐one‐out sensitivity analysis for ovarian cancer and coronary atherosclerosis. (B) Scatter plots demonstrating the robustness of the results from the analysis of causal relationships between ovarian cancer and coronary atherosclerosis using multiple methods.

To further investigate the potential bidirectional causal relationships, we performed reverse MR analyses with cancer as exposures and coronary atherosclerosis as the outcome. The results from these analyses indicated that there is no causal relationship between hepatic cancer or pancreatic cancer and coronary atherosclerosis. However, we found a nominally significant inverse association between ovariancancer and coronary atherosclerosis (OR = 0.97, 95*%*CI = 0.94–0.99, *p* value < 0.05) (Table S2), suggesting that genetic predisposition to ovarian cancer may be associated with slightly lower odds of coronary atherosclerosis. However, given the modest effect size (OR close to 1), this finding should be interpreted with caution. To validate these findings, we conducted additional heterogeneity tests **(**Table 3 **)**, horizontal pleiotropy tests **(**Table 4 **)**, and LOO sensitivity analyses (Figure 4A). The results from these tests, including scatter plots (Figure 4B), supported the robustness and reliability of our findings.

**Table 3:** Heterogeneity assessment of ovarian cancer and coronary atherosclerosis.

	id.exposure	id.outcome	Outcome	Exposure	Method	Q	Q_df	Q_pval
1	ebi‐a‐GCST90018888	finn‐b‐I9_CORATHER	Coronary atherosclerosis || id:finn‐b‐I9_CORATHER	|| id:ebi‐a‐GCST90018888	MR Egger	28.10618	28	0.458832
2	ebi‐a‐GCST90018888	finn‐b‐I9_CORATHER	Coronary atherosclerosis || id:finn‐b‐I9_CORATHER	|| id:ebi‐a‐GCST90018888	Inverse variance weighted	28.47526	29	0.492625

**Table 4:** Horizontal pleiotropy assessment of ovarian cancer and coronary atherosclerosis.

	id.exposure	id.outcome	Outcome	Exposure	egger_intercept	se	*p*
1	ebi‐a‐GCST90018888	finn‐b‐I9_CORATHER	Coronary atherosclerosis || id:finn‐b‐I9_CORATHER	|| id:ebi‐a‐GCST90018888	−0.00415	0.00685	0.549154

## 4. Discussion

Our study utilized MR to explore the causal relationships between coronary atherosclerosis and 23 types of cancer. Employing the IVW method, we identified causal associations between coronary atherosclerosis and three cancers: hepatic cancer, pancreatic cancer, and ovarian cancer. These results suggest that coronary atherosclerosis may influence the development of these cancers, offering insights into potential‐shared pathophysiological mechanisms. Importantly, all findings reported in this study are hypothesis‐generating in nature. Given the exploratory pan‐cancer design and the lack of multiple‐testing correction, these associations should not be interpreted as definitive causal evidence but rather as a foundation for future mechanistic and validation studies.

Our study provides evidence of causal relationships between coronary atherosclerosis and several types of cancer, notably hepatic cancer and pancreatic cancer. Using the IVW method, we found that coronary atherosclerosis is causally associated with a reduced risk of these cancers, with ORs of 0.87 (95*%*CI = 0.81–0.94, *p* value < 0.05) for hepatic cancer and 0.90 (95*%*CI = 0.83–0.98, *p* value < 0.05) for pancreatic cancer. These ORs being less than 1 indicate that coronary atherosclerosis may act as a protective factor against these cancers. This result is particularly intriguing, as numerous prior studies have indicated a shared pathophysiological mechanism between coronary atherosclerosis and cancer [14–16] However, certain epidemiological studies suggest a potential inverse correlation between coronary atherosclerosis and cancer, indicating that the mortality and incidence rates of coronary conditions increase with rising cholesterol levels, whereas the mortality and incidence rates of cancer exhibit a trend of inverse proportionality with cholesterol concentrations [17–19]. Additionally, some studies indicate that medications beneficial for patients with coronary artery disease, such as aspirin and statins, also confer protective effects for cancer patients [20–22]. Specifically, statins may exert anticancer properties in individuals with coronary atherosclerosis by lowering cholesterol levels and modulating inflammatory responses [23–25]. The inverse association we observed suggests that individuals with higher genetic predisposition to coronary atherosclerosis might have a lower risk of developing hepatic and pancreatic cancers. This finding is intriguing and counterintuitive, as one might expect that chronic inflammation and vascular damage associated with coronary atherosclerosis could increase cancer risk. However, our results imply a potentially complex interplay between coronary vascular pathology and cancer susceptibility, possibly involving shared genetic pathways or compensatory mechanisms that need further investigation. Our study reveals a significant causal relationship between ovarian cancer and coronary atherosclerosis, with an OR of 0.97 (95*%*CI = 0.94–0.99, *p* value < 0.05). The OR value of less than 1 indicates that ovarian cancer may act as a protective factor against coronary atherosclerosis. This finding suggests an inverse association, where individuals with a genetic predisposition to ovarian cancer might have a reduced risk of developing coronary atherosclerosis. The observed inverse relationship between ovarian cancer and coronary atherosclerosis raises intriguing questions about the underlying mechanisms.

This finding is intriguing and counterintuitive, as one might expect that the chronic inflammation and oxidative stress inherently associated with coronary atherosclerosis would promote, rather than reduce, carcinogenesis. To explain this paradox, several potential mechanisms must be considered. First, this inverse relationship may be driven by antagonistic (negative) pleiotropy. Genetic variants predisposing individuals to coronary atherosclerosis often involve significant alterations in lipid metabolism, such as elevated circulating LDL cholesterol. Although detrimental to vascular endothelium, specific systemic lipid profiles or the resulting metabolic reprogramming might simultaneously create a restrictive or unfavorable microenvironment for the initiation and proliferation of hepatic and pancreatic tumor cells.

The implications of our findings are significant for both clinical practice and future research [26–28]. If the suggestive inverse association between coronary atherosclerosis and hepatic/pancreatic cancers is functionally validated, it may serve as a hypothesis‐generating foundation for understanding shared metabolic pathways. It could be beneficial to explore whether therapeutic strategies targeting coronary atherosclerosis might also influence cancer risk or progression. Additionally, these results underscore the importance of considering shared genetic and pathophysiological mechanisms between cardiovascular diseases and cancers. Future research should aimed at further elucidating the biological pathways linking coronary atherosclerosis with cancer risk and validate these findings across diverse populations and study designs. The identification of a suggestive inverse association between ovarian cancer and coronary atherosclerosis provides hypothesis‐generating insights. Although direct clinical applications are premature at this stage, if validated, it could eventually inform new perspectives on managing cardiovascular risk in patients with ovarian cancer and vice versa [29–31]. For example, understanding how ovarian cancer affects cardiovascular health could reveal new strategies for cardiovascular risk assessment and management in oncology patients. Additionally, these findings suggest that cancer‐related factors or treatments might influence cardiovascular health in complex ways. Oncologists and cardiologists may need to consider these interactions when developing comprehensive care plans for patients with ovarian cancer.

In summary, our study offers novel insights into the complex directional relationships between coronary atherosclerosis and cancer. Through forward MR, we identified a protective association of coronary atherosclerosis against hepatic and pancreatic cancers. Through reverse MR, we provided new evidence of an inverse causal relationship, where ovarian cancer exerts a protective effect on coronary atherosclerosis. These findings contribute to a deeper understanding of the interplay between cardiovascular and cancer diseases, suggesting new avenues for integrated prevention and treatment strategies. They underscore the necessity for further research to unravel the complex interactions between cancer and cardiovascular conditions, potentially paving the way for innovative risk management and therapeutic approaches. Nevertheless, given the exploratory nature of this study and the modest effect sizes observed, these findings should be considered hypothesis‐generating rather than clinically actionable. Independent replication in larger, more diverse cohorts and functional studies are essential before any translational implications can be drawn.

## 5. Conclusion

In conclusion, this two‐sample bidirectional MR study provides hypothesis‐generating genetic evidence for potential links between coronary atherosclerosis and selected cancer types. In the forward MR analysis, genetically predicted coronary atherosclerosis was associated with lower odds of pancreatic cancer and hepatic cancer, though these associations were modest and require replication. In the reverse MR analysis, genetic predisposition to ovarian cancer showed a modest inverse association with coronary atherosclerosis. These findings indicate a potentially complex and bidirectional genetic interplay between cardiovascular disease and cancer, which may involve shared biological pathways related to lipid metabolism, inflammation, oxidative stress, and vascular or tumor microenvironmental regulation. However, given the exploratory nature of this pan‐cancer MR analysis and the absence of multiple testing correction, these associations should be interpreted as hypothesis‐generating rather than definitive causal evidence. Further validation using larger GWAS datasets, sex‐stratified analyses, multiethnic populations, and functional experimental studies is warranted to clarify the underlying mechanisms and potential clinical implications of the observed cardiovascularÃ¢â‚¬â€œoncological connections.

## 6. Limitations

Although our study provides valuable insights into the genetic associations between coronary atherosclerosis and cancer using MR, several limitations should be acknowledged. First, the MR approach relies on the assumption that genetic variants used as IVs are strongly associated with the exposure and do not influence the outcome through pathways other than the exposure (no horizontal pleiotropy). Although we employed MR‐Egger regression and sensitivity analyses to address potential pleiotropy, residual confounding cannot be entirely ruled out. Second, the GWAS data used in this study were primarily derived from European populations, which may limit the generalizability of our findings to other ethnic groups. Third, the sample sizes for certain cancer types were relatively small, potentially reducing the statistical power to detect causal relationships. Fourth, our analysis focused on genetic predisposition rather than direct measurements of environmental or lifestyle factors, which may also play significant roles in the development of both coronary atherosclerosis and cancer. Finally, although MR provides evidence of causality, it does not elucidate the underlying biological mechanisms, warranting further experimental and clinical studies to validate and explore these findings in greater depth. We did not apply multiple testing correction methods (such as Bonferroni or false discovery rate) to our statistical analyses. Given the exploratory nature of this comprehensive pan‐cancer MR study, we opted to report nominally significant associations (*p* < 0.05) to minimize the risk of Type II errors and to avoid overlooking potential biological signals that could inform future research. Consequently, the lack of correction may inflate the Type I error rate. Therefore, the causal relationships identified in this study should be interpreted with caution and regarded as suggestive evidence, which warrants further validation through independent, large‐scale cohorts and experimental studies. The GWAS sample sizes for certain cancer types (e.g., testicular cancer and mesothelioma) were relatively small, which inherently limits the statistical power of our MR analyses. Because we did not perform post hoc power calculations, we cannot rule out the possibility of false negatives for these specific outcomes. Consequently, the null findings observed for these cancers may be attributed to insufficient statistical power rather than a true absence of a genetic causal relationship. Future MR studies leveraging larger, more comprehensive GWAS meta‐analyses are warranted to definitively confirm or refute these null associations. Our analysis included sex‐specific malignancies (e.g., ovarian and testicular cancers) while utilizing a sex‐combined GWAS dataset for coronary atherosclerosis. This sex imbalance inherently introduces potential confounding and statistical noise. Applying genetic instruments derived from a sex‐specific cohort to a sex‐combined outcome may dilute the true causal effect size or obscure sex‐specific pleiotropic pathways. Because sex‐stratified GWAS summary statistics for coronary atherosclerosis were not readily available for our analysis, we were unable to perform sex‐stratified MR. Therefore, the findings regarding sex‐specific cancers should be interpreted with caution, and future studies utilizing sex‐specific cardiovascular GWAS datasets are essential to validate these associations.

## Author Contributions

Yanan Fan and Wei Geng conceived and designed the study. Analysis were conducted by Yanan Fan, Miaomiao Liu, Lifei Zhang, and Fei Liu. Jiantao Dong, Mengyang Zhang, Wenjing Yao, and Lixiao Zhang performed the data analysis. The manuscript was drafted by Yanan Fan, Yun Sun, and Wei Geng. Yanan Fan, Miaomiao Liu, and Lifei Zhang have contributed equally to this work.

## Funding

This research received financial support from the Hebei Provincial Medical Science Research Project Plan (Grant No. 20231478) and the 2024 Clinical Medicine Excellent Talent Training Project, funded by the Hebei Provincial Government (Grant Nos. ZF2024017 and ZF2024021).

## Disclosure

All authors reviewed and approved the final version of the manuscript.

## Ethics Statement

As this study used publicly available summary‐level GWAS data, no institutional ethical approval was required. Furthermore, no individual‐level data were accessed during the course of this research. No clinical trial registration is associated with this work.

## Consent

The authors confirm that this work is not under review by any other publication. Furthermore, all individuals listed as coauthors have been informed of and have approved the submission of this manuscript.

## Conflicts of Interest

The authors declare no conflicts of interest.

## Supporting Information

Additional supporting information can be found online in the Supporting Information section.

## Supporting information


**Supporting Information 1** Figure S1: Mendelian randomization leave‐one‐out sensitivity analysis for coronary atherosclerosis and hepatic cancer. Figure S2: Mendelian randomization leave‐one‐out sensitivity analysis for coronary atherosclerosis and lung cancer. Figure S3: Mendelian randomization leave‐one‐out sensitivity analysis for coronary atherosclerosis and pancreatic cancer.

**Supporting Information 2** Table S1: Causal relationship analysis between coronary atherosclerosis and 23 types of cancer. Table S2: Reverse Mendelian Randomization analysis assessing the causality between coronary atherosclerosis and 23 types of cancer.

## Data Availability

All datasets generated and analyzed during this study are openly accessible from the GWAS Catalog (https://gwas.mrcieu.ac.uk/). Any additional questions regarding the data should be directed to the authors.
