# Supplementary material for: Genetic Links Between Cancer and Coronary Atherosclerosis: A Mendelian Randomization Analysis
Source: Hum Mutat. 2026 Jul 8;2026:5997499. doi: 10.1155/humu/5997499 (PMC13346356; doi:10.1155/humu/5997499)

**Figure S1:**

Mendelian Randomization Leave-One-Out Sensitivity Analysis for Coronary Atherosclerosis and Hepatic Cancer.


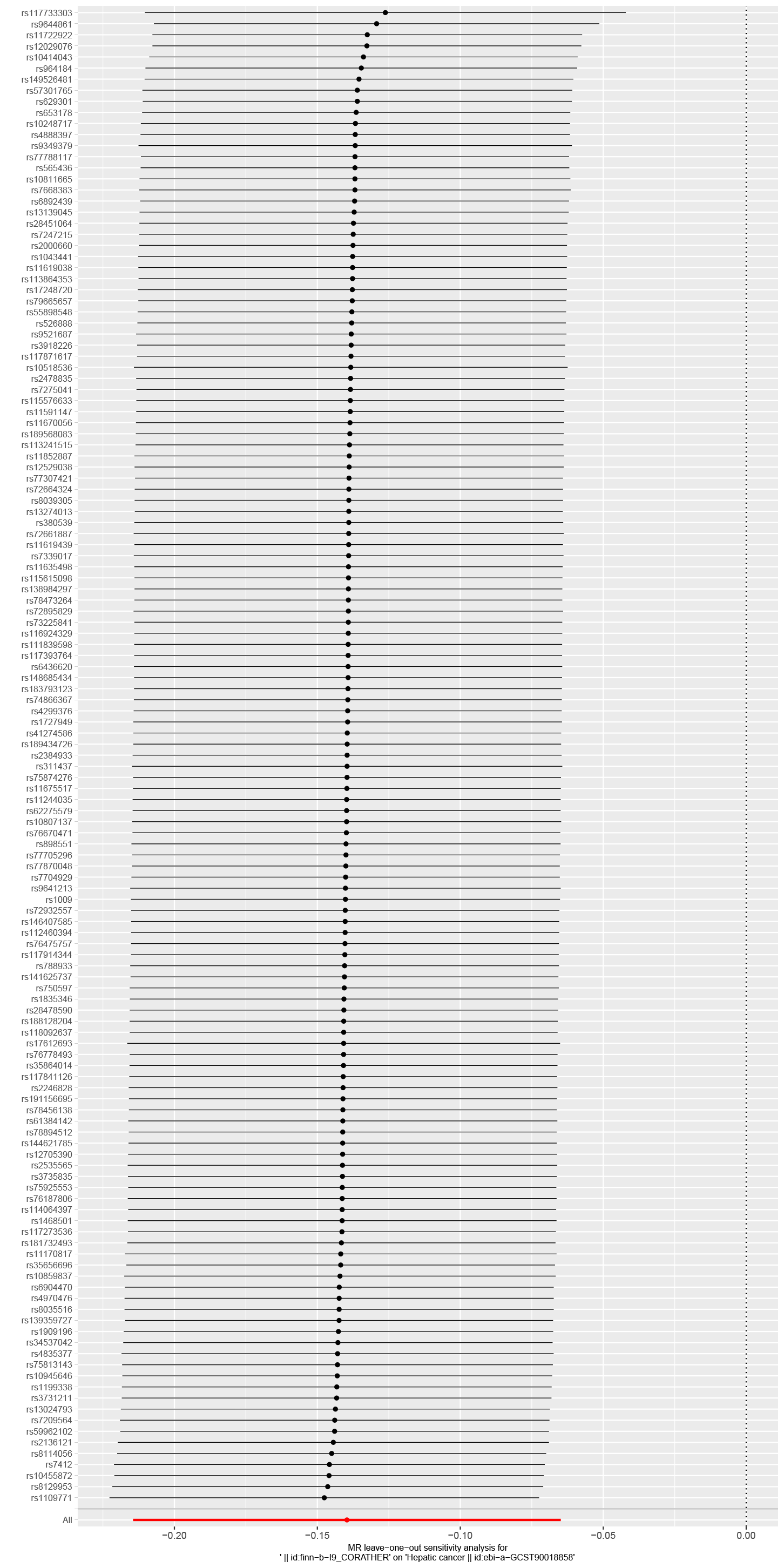


**Figure S2:**

Mendelian Randomization Leave-One-Out Sensitivity Analysis for Coronary Atherosclerosis and Lung Cancer.


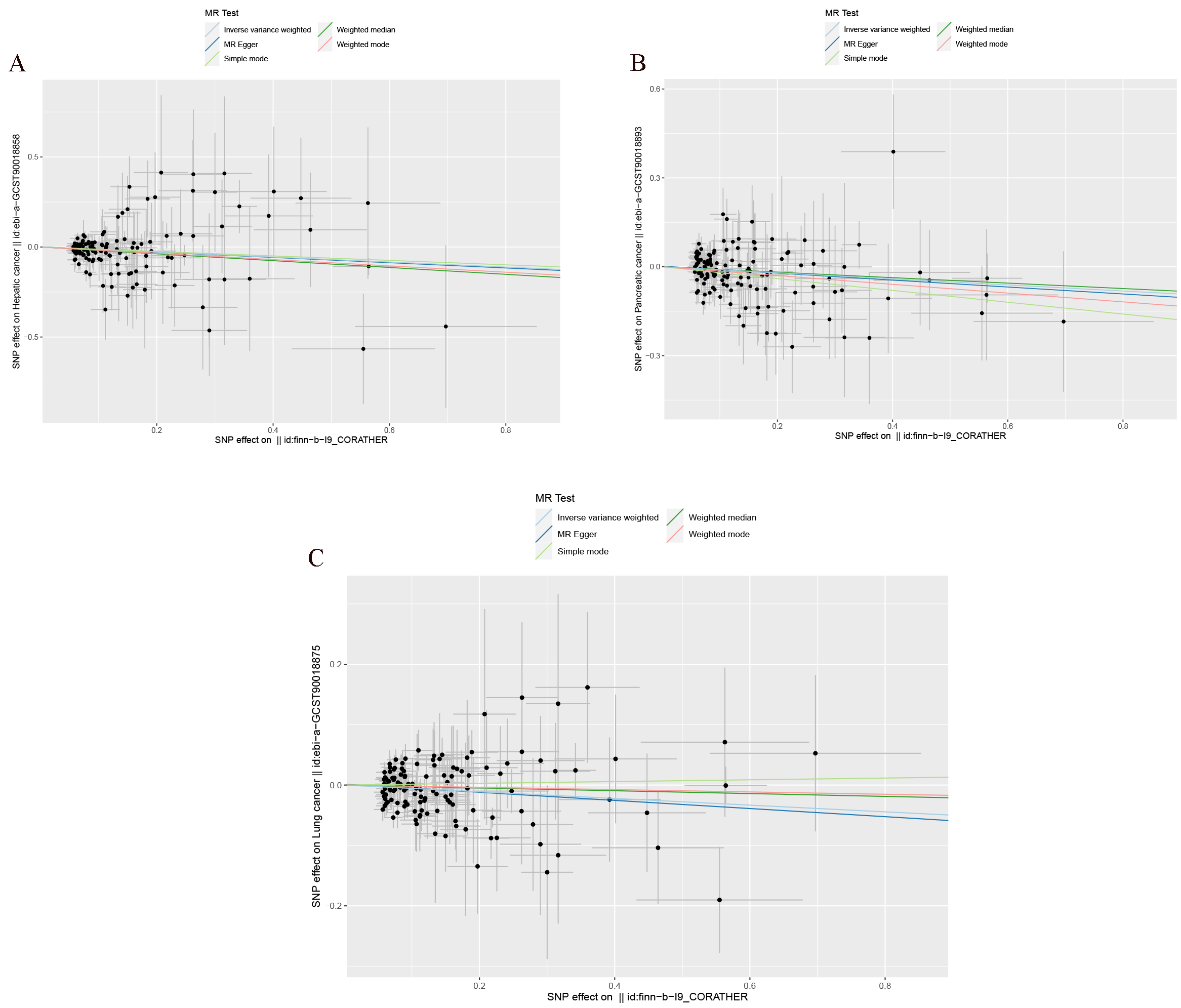


**Figure S3:**

Mendelian Randomization Leave-One-Out Sensitivity Analysis for Coronary Atherosclerosis and ancreatic cancer.


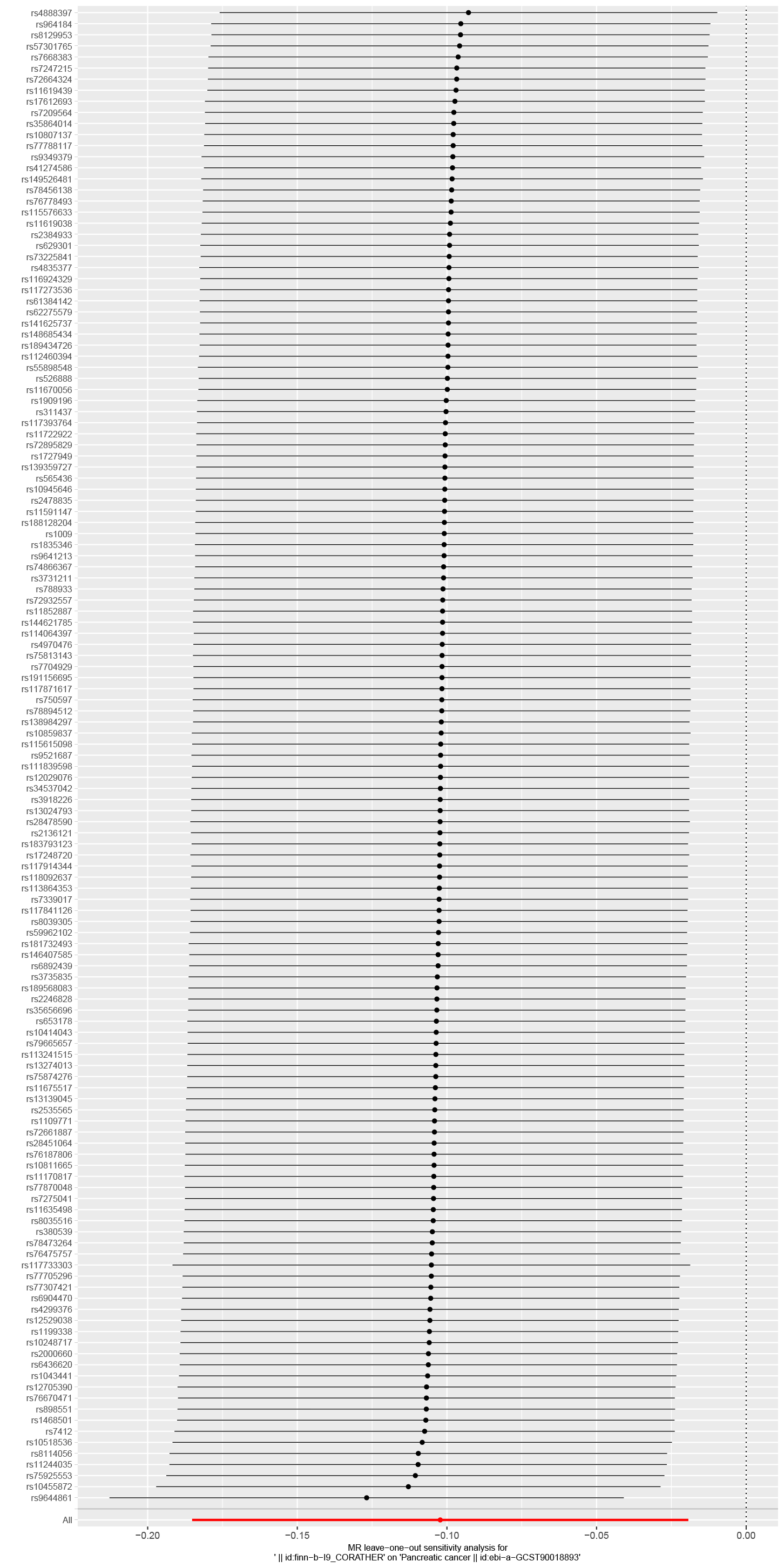

Supplement: Supplementary file 1 — Supporting Information 1 Figure S1: Mendelian randomization leave‐one‐out sensitivity analysis for coronary atherosclerosis and hepatic cancer. Figure S2: Mendelian randomization leave‐one‐out sensitivity analysis for coronary atherosclerosis and lung cancer. Figure S3: Mendelian randomization leave‐one‐out sensitivity analysis for coronary atherosclerosis and pancreatic cancer. [file HUMU-2026-5997499-s002.docx]
