# Supplementary material for: Genetic Links Between Cancer and Coronary Atherosclerosis: A Mendelian Randomization Analysis
Source: Hum Mutat. 2026 Jul 8;2026:5997499. doi: 10.1155/humu/5997499 (PMC13346356; doi:10.1155/humu/5997499)
Supplement: Supplementary file 2 — Supporting Information 2 Table S1: Causal relationship analysis between coronary atherosclerosis and 23 types of cancer. Table S2: Reverse Mendelian Randomization analysis assessing the causality between coronary atherosclerosis and 23 types of cancer. [file HUMU-2026-5997499-s001.docx]

**Table S1:** Causal Relationship Analysis Between Coronary Atherosclerosis and 23 Types of Cancer.

|  | id.exposure | id.outcome | outcome | exposure | method | nsnp | b | se | pval | lo_ci | up_ci | or | or_lci95 | or_uci95 |
| --- | --- | --- | --- | --- | --- | --- | --- | --- | --- | --- | --- | --- | --- | --- |
| 1 | finn-b-I9_CORATHER | ebi-a-GCST004744 | Lung adenocarcinoma \|\| id:ebi-a-GCST004744 | \|\| id:finn-b-I9_CORATHER | MR Egger | 125 | -0.06301 | 0.049251 | 0.203203 | -0.15954 | 0.033526 | 0.938938 | 0.852538 | 1.034094 |
| 2 | finn-b-I9_CORATHER | ebi-a-GCST004744 | Lung adenocarcinoma \|\| id:ebi-a-GCST004744 | \|\| id:finn-b-I9_CORATHER | Weighted median | 125 | 0.0082 | 0.033191 | 0.804875 | -0.05685 | 0.073254 | 1.008233 | 0.944732 | 1.076003 |
| 3 | finn-b-I9_CORATHER | ebi-a-GCST004744 | Lung adenocarcinoma \|\| id:ebi-a-GCST004744 | \|\| id:finn-b-I9_CORATHER | Inverse variance weighted | 125 | -0.05253 | 0.024303 | 0.030675 | -0.10016 | -0.00489 | 0.94883 | 0.904692 | 0.995121 |
| 4 | finn-b-I9_CORATHER | ebi-a-GCST004744 | Lung adenocarcinoma \|\| id:ebi-a-GCST004744 | \|\| id:finn-b-I9_CORATHER | Simple mode | 125 | 0.067684 | 0.080841 | 0.404065 | -0.09076 | 0.226132 | 1.070027 | 0.913233 | 1.253741 |
| 5 | finn-b-I9_CORATHER | ebi-a-GCST004744 | Lung adenocarcinoma \|\| id:ebi-a-GCST004744 | \|\| id:finn-b-I9_CORATHER | Weighted mode | 125 | 0.033307 | 0.070317 | 0.636569 | -0.10451 | 0.171128 | 1.033868 | 0.900762 | 1.186642 |
| 6 | finn-b-I9_CORATHER | ebi-a-GCST90018799 | Breast cancer \|\| id:ebi-a-GCST90018799 | \|\| id:finn-b-I9_CORATHER | MR Egger | 135 | -0.0306 | 0.030293 | 0.314329 | -0.08997 | 0.028778 | 0.969867 | 0.913959 | 1.029196 |
| 7 | finn-b-I9_CORATHER | ebi-a-GCST90018799 | Breast cancer \|\| id:ebi-a-GCST90018799 | \|\| id:finn-b-I9_CORATHER | Weighted median | 135 | -0.03611 | 0.020102 | 0.072443 | -0.07551 | 0.00329 | 0.964535 | 0.927272 | 1.003296 |
| 8 | finn-b-I9_CORATHER | ebi-a-GCST90018799 | Breast cancer \|\| id:ebi-a-GCST90018799 | \|\| id:finn-b-I9_CORATHER | Inverse variance weighted | 135 | -0.0061 | 0.015638 | 0.696356 | -0.03675 | 0.024548 | 0.993916 | 0.963914 | 1.024852 |
| 9 | finn-b-I9_CORATHER | ebi-a-GCST90018799 | Breast cancer \|\| id:ebi-a-GCST90018799 | \|\| id:finn-b-I9_CORATHER | Simple mode | 135 | -0.09182 | 0.050193 | 0.069575 | -0.1902 | 0.006559 | 0.912271 | 0.826797 | 1.006581 |
| 10 | finn-b-I9_CORATHER | ebi-a-GCST90018799 | Breast cancer \|\| id:ebi-a-GCST90018799 | \|\| id:finn-b-I9_CORATHER | Weighted mode | 135 | -0.08847 | 0.035803 | 0.014724 | -0.15865 | -0.0183 | 0.915327 | 0.853297 | 0.981867 |
| 11 | finn-b-I9_CORATHER | ebi-a-GCST90018803 | Hepatic bile duct cancer \|\| id:ebi-a-GCST90018803 | \|\| id:finn-b-I9_CORATHER | MR Egger | 135 | 0.074608 | 0.105849 | 0.482133 | -0.13286 | 0.282072 | 1.077462 | 0.875592 | 1.325874 |
| 12 | finn-b-I9_CORATHER | ebi-a-GCST90018803 | Hepatic bile duct cancer \|\| id:ebi-a-GCST90018803 | \|\| id:finn-b-I9_CORATHER | Weighted median | 135 | -0.02484 | 0.082532 | 0.763396 | -0.18661 | 0.136919 | 0.975462 | 0.829769 | 1.146735 |
| 13 | finn-b-I9_CORATHER | ebi-a-GCST90018803 | Hepatic bile duct cancer \|\| id:ebi-a-GCST90018803 | \|\| id:finn-b-I9_CORATHER | Inverse variance weighted | 135 | -0.05559 | 0.055121 | 0.313184 | -0.16363 | 0.052444 | 0.945924 | 0.849056 | 1.053844 |
| 14 | finn-b-I9_CORATHER | ebi-a-GCST90018803 | Hepatic bile duct cancer \|\| id:ebi-a-GCST90018803 | \|\| id:finn-b-I9_CORATHER | Simple mode | 135 | -0.37847 | 0.222438 | 0.091174 | -0.81445 | 0.057506 | 0.684907 | 0.442883 | 1.059192 |
| 15 | finn-b-I9_CORATHER | ebi-a-GCST90018803 | Hepatic bile duct cancer \|\| id:ebi-a-GCST90018803 | \|\| id:finn-b-I9_CORATHER | Weighted mode | 135 | 0.138057 | 0.149263 | 0.356668 | -0.1545 | 0.430613 | 1.148041 | 0.856844 | 1.538201 |
| 16 | finn-b-I9_CORATHER | ebi-a-GCST90018808 | Colorectal cancer \|\| id:ebi-a-GCST90018808 | \|\| id:finn-b-I9_CORATHER | MR Egger | 135 | -0.01794 | 0.039332 | 0.649022 | -0.09503 | 0.05915 | 0.982218 | 0.909343 | 1.060934 |
| 17 | finn-b-I9_CORATHER | ebi-a-GCST90018808 | Colorectal cancer \|\| id:ebi-a-GCST90018808 | \|\| id:finn-b-I9_CORATHER | Weighted median | 135 | -0.05298 | 0.027763 | 0.056349 | -0.1074 | 0.001435 | 0.948398 | 0.898169 | 1.001436 |
| 18 | finn-b-I9_CORATHER | ebi-a-GCST90018808 | Colorectal cancer \|\| id:ebi-a-GCST90018808 | \|\| id:finn-b-I9_CORATHER | Inverse variance weighted | 135 | -0.07109 | 0.020692 | 0.000591 | -0.11165 | -0.03054 | 0.931376 | 0.89436 | 0.969925 |
| 19 | finn-b-I9_CORATHER | ebi-a-GCST90018808 | Colorectal cancer \|\| id:ebi-a-GCST90018808 | \|\| id:finn-b-I9_CORATHER | Simple mode | 135 | -0.16907 | 0.080943 | 0.038625 | -0.32772 | -0.01042 | 0.844452 | 0.720567 | 0.989635 |
| 20 | finn-b-I9_CORATHER | ebi-a-GCST90018808 | Colorectal cancer \|\| id:ebi-a-GCST90018808 | \|\| id:finn-b-I9_CORATHER | Weighted mode | 135 | -0.01919 | 0.041814 | 0.646989 | -0.10115 | 0.062763 | 0.980991 | 0.9038 | 1.064775 |
| 21 | finn-b-I9_CORATHER | ebi-a-GCST90018817 | Cervical cancer \|\| id:ebi-a-GCST90018817 | \|\| id:finn-b-I9_CORATHER | MR Egger | 134 | -0.02099 | 0.081779 | 0.797829 | -0.18128 | 0.139297 | 0.979228 | 0.834203 | 1.149465 |
| 22 | finn-b-I9_CORATHER | ebi-a-GCST90018817 | Cervical cancer \|\| id:ebi-a-GCST90018817 | \|\| id:finn-b-I9_CORATHER | Weighted median | 134 | -0.03352 | 0.072343 | 0.643093 | -0.17532 | 0.108271 | 0.967033 | 0.839192 | 1.114349 |
| 23 | finn-b-I9_CORATHER | ebi-a-GCST90018817 | Cervical cancer \|\| id:ebi-a-GCST90018817 | \|\| id:finn-b-I9_CORATHER | Inverse variance weighted | 134 | 0.001286 | 0.042607 | 0.975913 | -0.08222 | 0.084796 | 1.001287 | 0.921066 | 1.088495 |
| 24 | finn-b-I9_CORATHER | ebi-a-GCST90018817 | Cervical cancer \|\| id:ebi-a-GCST90018817 | \|\| id:finn-b-I9_CORATHER | Simple mode | 134 | 0.150777 | 0.128263 | 0.241885 | -0.10062 | 0.402172 | 1.162737 | 0.904277 | 1.495069 |
| 25 | finn-b-I9_CORATHER | ebi-a-GCST90018817 | Cervical cancer \|\| id:ebi-a-GCST90018817 | \|\| id:finn-b-I9_CORATHER | Weighted mode | 134 | -0.01538 | 0.084618 | 0.856003 | -0.18124 | 0.150466 | 0.984733 | 0.834238 | 1.162376 |
| 26 | finn-b-I9_CORATHER | ebi-a-GCST90018841 | Esophageal cancer \|\| id:ebi-a-GCST90018841 | \|\| id:finn-b-I9_CORATHER | MR Egger | 135 | -0.11886 | 0.07065 | 0.094842 | -0.25734 | 0.019614 | 0.887931 | 0.773109 | 1.019808 |
| 27 | finn-b-I9_CORATHER | ebi-a-GCST90018841 | Esophageal cancer \|\| id:ebi-a-GCST90018841 | \|\| id:finn-b-I9_CORATHER | Weighted median | 135 | -0.09017 | 0.069252 | 0.192913 | -0.2259 | 0.045567 | 0.913778 | 0.797796 | 1.046622 |
| 28 | finn-b-I9_CORATHER | ebi-a-GCST90018841 | Esophageal cancer \|\| id:ebi-a-GCST90018841 | \|\| id:finn-b-I9_CORATHER | Inverse variance weighted | 135 | -0.01885 | 0.037095 | 0.611315 | -0.09156 | 0.053854 | 0.981325 | 0.91251 | 1.055331 |
| 29 | finn-b-I9_CORATHER | ebi-a-GCST90018841 | Esophageal cancer \|\| id:ebi-a-GCST90018841 | \|\| id:finn-b-I9_CORATHER | Simple mode | 135 | -0.02577 | 0.141311 | 0.85558 | -0.30274 | 0.251201 | 0.97456 | 0.738793 | 1.285568 |
| 30 | finn-b-I9_CORATHER | ebi-a-GCST90018841 | Esophageal cancer \|\| id:ebi-a-GCST90018841 | \|\| id:finn-b-I9_CORATHER | Weighted mode | 135 | -0.1097 | 0.072679 | 0.133549 | -0.25215 | 0.032749 | 0.896101 | 0.777126 | 1.033291 |
| 31 | finn-b-I9_CORATHER | ebi-a-GCST90018849 | Gastric cancer \|\| id:ebi-a-GCST90018849 | \|\| id:finn-b-I9_CORATHER | MR Egger | 135 | -0.01153 | 0.041586 | 0.781994 | -0.09304 | 0.069978 | 0.988535 | 0.911157 | 1.072485 |
| 32 | finn-b-I9_CORATHER | ebi-a-GCST90018849 | Gastric cancer \|\| id:ebi-a-GCST90018849 | \|\| id:finn-b-I9_CORATHER | Weighted median | 135 | -0.0093 | 0.036426 | 0.798575 | -0.08069 | 0.062099 | 0.990747 | 0.922479 | 1.064068 |
| 33 | finn-b-I9_CORATHER | ebi-a-GCST90018849 | Gastric cancer \|\| id:ebi-a-GCST90018849 | \|\| id:finn-b-I9_CORATHER | Inverse variance weighted | 135 | -0.04183 | 0.021844 | 0.055517 | -0.08464 | 0.000987 | 0.959035 | 0.918841 | 1.000988 |
| 34 | finn-b-I9_CORATHER | ebi-a-GCST90018849 | Gastric cancer \|\| id:ebi-a-GCST90018849 | \|\| id:finn-b-I9_CORATHER | Simple mode | 135 | -0.05466 | 0.066457 | 0.412267 | -0.18491 | 0.075596 | 0.946808 | 0.831175 | 1.078527 |
| 35 | finn-b-I9_CORATHER | ebi-a-GCST90018849 | Gastric cancer \|\| id:ebi-a-GCST90018849 | \|\| id:finn-b-I9_CORATHER | Weighted mode | 135 | -0.02424 | 0.033636 | 0.472449 | -0.09016 | 0.04169 | 0.976056 | 0.913784 | 1.042571 |
| 36 | finn-b-I9_CORATHER | ebi-a-GCST90018858 | Hepatic cancer \|\| id:ebi-a-GCST90018858 | \|\| id:finn-b-I9_CORATHER | MR Egger | 135 | -0.14666 | 0.072833 | 0.046061 | -0.28942 | -0.00391 | 0.863585 | 0.748701 | 0.996098 |
| 37 | finn-b-I9_CORATHER | ebi-a-GCST90018858 | Hepatic cancer \|\| id:ebi-a-GCST90018858 | \|\| id:finn-b-I9_CORATHER | Weighted median | 135 | -0.18972 | 0.069469 | 0.006314 | -0.32588 | -0.05356 | 0.827191 | 0.721893 | 0.947849 |
| 38 | finn-b-I9_CORATHER | ebi-a-GCST90018858 | Hepatic cancer \|\| id:ebi-a-GCST90018858 | \|\| id:finn-b-I9_CORATHER | Inverse variance weighted | 135 | -0.13965 | 0.038164 | 0.000253 | -0.21445 | -0.06485 | 0.869661 | 0.806983 | 0.937208 |
| 39 | finn-b-I9_CORATHER | ebi-a-GCST90018858 | Hepatic cancer \|\| id:ebi-a-GCST90018858 | \|\| id:finn-b-I9_CORATHER | Simple mode | 135 | -0.12393 | 0.110566 | 0.264351 | -0.34064 | 0.09278 | 0.883443 | 0.711317 | 1.09722 |
| 40 | finn-b-I9_CORATHER | ebi-a-GCST90018858 | Hepatic cancer \|\| id:ebi-a-GCST90018858 | \|\| id:finn-b-I9_CORATHER | Weighted mode | 135 | -0.1751 | 0.061881 | 0.005377 | -0.29639 | -0.05382 | 0.83937 | 0.743497 | 0.947606 |
| 41 | finn-b-I9_CORATHER | ebi-a-GCST90018875 | Lung cancer \|\| id:ebi-a-GCST90018875 | \|\| id:finn-b-I9_CORATHER | MR Egger | 135 | -0.06736 | 0.039981 | 0.094362 | -0.14573 | 0.011001 | 0.934856 | 0.864394 | 1.011062 |
| 42 | finn-b-I9_CORATHER | ebi-a-GCST90018875 | Lung cancer \|\| id:ebi-a-GCST90018875 | \|\| id:finn-b-I9_CORATHER | Weighted median | 135 | -0.02351 | 0.035209 | 0.504323 | -0.09252 | 0.0455 | 0.976765 | 0.911633 | 1.046551 |
| 43 | finn-b-I9_CORATHER | ebi-a-GCST90018875 | Lung cancer \|\| id:ebi-a-GCST90018875 | \|\| id:finn-b-I9_CORATHER | Inverse variance weighted | 135 | -0.05548 | 0.02072 | 0.007415 | -0.09609 | -0.01487 | 0.946031 | 0.908382 | 0.985241 |
| 44 | finn-b-I9_CORATHER | ebi-a-GCST90018875 | Lung cancer \|\| id:ebi-a-GCST90018875 | \|\| id:finn-b-I9_CORATHER | Simple mode | 135 | 0.014483 | 0.082417 | 0.86077 | -0.14705 | 0.17602 | 1.014589 | 0.863248 | 1.192462 |
| 45 | finn-b-I9_CORATHER | ebi-a-GCST90018875 | Lung cancer \|\| id:ebi-a-GCST90018875 | \|\| id:finn-b-I9_CORATHER | Weighted mode | 135 | -0.01907 | 0.049878 | 0.702834 | -0.11683 | 0.078693 | 0.981111 | 0.889736 | 1.081872 |
| 46 | finn-b-I9_CORATHER | ebi-a-GCST90018888 | Ovarian cancer \|\| id:ebi-a-GCST90018888 | \|\| id:finn-b-I9_CORATHER | MR Egger | 134 | 0.043127 | 0.06878 | 0.53173 | -0.09168 | 0.177936 | 1.04407 | 0.912394 | 1.194749 |
| 47 | finn-b-I9_CORATHER | ebi-a-GCST90018888 | Ovarian cancer \|\| id:ebi-a-GCST90018888 | \|\| id:finn-b-I9_CORATHER | Weighted median | 134 | 0.035477 | 0.060685 | 0.558807 | -0.08347 | 0.15442 | 1.036114 | 0.919923 | 1.166981 |
| 48 | finn-b-I9_CORATHER | ebi-a-GCST90018888 | Ovarian cancer \|\| id:ebi-a-GCST90018888 | \|\| id:finn-b-I9_CORATHER | Inverse variance weighted | 134 | 0.038983 | 0.035637 | 0.274009 | -0.03087 | 0.108832 | 1.039753 | 0.969605 | 1.114975 |
| 49 | finn-b-I9_CORATHER | ebi-a-GCST90018888 | Ovarian cancer \|\| id:ebi-a-GCST90018888 | \|\| id:finn-b-I9_CORATHER | Simple mode | 134 | -0.06652 | 0.129389 | 0.608041 | -0.32012 | 0.187084 | 0.935647 | 0.726063 | 1.205729 |
| 50 | finn-b-I9_CORATHER | ebi-a-GCST90018888 | Ovarian cancer \|\| id:ebi-a-GCST90018888 | \|\| id:finn-b-I9_CORATHER | Weighted mode | 134 | -0.00306 | 0.080309 | 0.969699 | -0.16046 | 0.154349 | 0.996948 | 0.851751 | 1.166898 |
| 51 | finn-b-I9_CORATHER | ebi-a-GCST90018893 | Pancreatic cancer \|\| id:ebi-a-GCST90018893 | \|\| id:finn-b-I9_CORATHER | MR Egger | 135 | -0.1171 | 0.082415 | 0.157689 | -0.27864 | 0.044431 | 0.889494 | 0.756816 | 1.045433 |
| 52 | finn-b-I9_CORATHER | ebi-a-GCST90018893 | Pancreatic cancer \|\| id:ebi-a-GCST90018893 | \|\| id:finn-b-I9_CORATHER | Weighted median | 135 | -0.09183 | 0.074973 | 0.220612 | -0.23878 | 0.055112 | 0.912256 | 0.787587 | 1.056659 |
| 53 | finn-b-I9_CORATHER | ebi-a-GCST90018893 | Pancreatic cancer \|\| id:ebi-a-GCST90018893 | \|\| id:finn-b-I9_CORATHER | Inverse variance weighted | 135 | -0.10224 | 0.042297 | 0.015646 | -0.18514 | -0.01933 | 0.902817 | 0.83099 | 0.980853 |
| 54 | finn-b-I9_CORATHER | ebi-a-GCST90018893 | Pancreatic cancer \|\| id:ebi-a-GCST90018893 | \|\| id:finn-b-I9_CORATHER | Simple mode | 135 | -0.19948 | 0.153327 | 0.19549 | -0.5 | 0.101042 | 0.819157 | 0.606531 | 1.106323 |
| 55 | finn-b-I9_CORATHER | ebi-a-GCST90018893 | Pancreatic cancer \|\| id:ebi-a-GCST90018893 | \|\| id:finn-b-I9_CORATHER | Weighted mode | 135 | -0.14811 | 0.117955 | 0.211416 | -0.3793 | 0.083077 | 0.862333 | 0.684337 | 1.086626 |
| 56 | finn-b-I9_CORATHER | ebi-a-GCST90018905 | Prostate cancer \|\| id:ebi-a-GCST90018905 | \|\| id:finn-b-I9_CORATHER | MR Egger | 135 | -0.01249 | 0.034808 | 0.720235 | -0.08072 | 0.055731 | 0.987585 | 0.922455 | 1.057313 |
| 57 | finn-b-I9_CORATHER | ebi-a-GCST90018905 | Prostate cancer \|\| id:ebi-a-GCST90018905 | \|\| id:finn-b-I9_CORATHER | Weighted median | 135 | -0.01832 | 0.024889 | 0.461789 | -0.0671 | 0.030467 | 0.98185 | 0.935102 | 1.030936 |
| 58 | finn-b-I9_CORATHER | ebi-a-GCST90018905 | Prostate cancer \|\| id:ebi-a-GCST90018905 | \|\| id:finn-b-I9_CORATHER | Inverse variance weighted | 135 | -0.00941 | 0.017879 | 0.598696 | -0.04445 | 0.025634 | 0.990635 | 0.95652 | 1.025966 |
| 59 | finn-b-I9_CORATHER | ebi-a-GCST90018905 | Prostate cancer \|\| id:ebi-a-GCST90018905 | \|\| id:finn-b-I9_CORATHER | Simple mode | 135 | -0.03841 | 0.053112 | 0.470766 | -0.14251 | 0.065684 | 0.962314 | 0.867176 | 1.067889 |
| 60 | finn-b-I9_CORATHER | ebi-a-GCST90018905 | Prostate cancer \|\| id:ebi-a-GCST90018905 | \|\| id:finn-b-I9_CORATHER | Weighted mode | 135 | -0.02513 | 0.033411 | 0.453349 | -0.09061 | 0.040359 | 0.975187 | 0.913372 | 1.041185 |
| 61 | finn-b-I9_CORATHER | ebi-a-GCST90018921 | Skin cancer \|\| id:ebi-a-GCST90018921 | \|\| id:finn-b-I9_CORATHER | MR Egger | 135 | -0.02542 | 0.023996 | 0.29136 | -0.07245 | 0.021612 | 0.9749 | 0.930111 | 1.021847 |
| 62 | finn-b-I9_CORATHER | ebi-a-GCST90018921 | Skin cancer \|\| id:ebi-a-GCST90018921 | \|\| id:finn-b-I9_CORATHER | Weighted median | 135 | -0.03293 | 0.018227 | 0.070784 | -0.06866 | 0.002791 | 0.967602 | 0.933645 | 1.002795 |
| 63 | finn-b-I9_CORATHER | ebi-a-GCST90018921 | Skin cancer \|\| id:ebi-a-GCST90018921 | \|\| id:finn-b-I9_CORATHER | Inverse variance weighted | 135 | -0.02408 | 0.012485 | 0.053756 | -0.04855 | 0.00039 | 0.976207 | 0.952609 | 1.00039 |
| 64 | finn-b-I9_CORATHER | ebi-a-GCST90018921 | Skin cancer \|\| id:ebi-a-GCST90018921 | \|\| id:finn-b-I9_CORATHER | Simple mode | 135 | -0.04363 | 0.043448 | 0.317104 | -0.12879 | 0.041528 | 0.957309 | 0.879162 | 1.042403 |
| 65 | finn-b-I9_CORATHER | ebi-a-GCST90018921 | Skin cancer \|\| id:ebi-a-GCST90018921 | \|\| id:finn-b-I9_CORATHER | Weighted mode | 135 | -0.04363 | 0.036934 | 0.239592 | -0.11602 | 0.028762 | 0.957309 | 0.890457 | 1.02918 |
| 66 | finn-b-I9_CORATHER | ebi-a-GCST90018929 | Thyroid cancer \|\| id:ebi-a-GCST90018929 | \|\| id:finn-b-I9_CORATHER | MR Egger | 135 | 0.096372 | 0.092009 | 0.296808 | -0.08397 | 0.276709 | 1.101168 | 0.919462 | 1.318783 |
| 67 | finn-b-I9_CORATHER | ebi-a-GCST90018929 | Thyroid cancer \|\| id:ebi-a-GCST90018929 | \|\| id:finn-b-I9_CORATHER | Weighted median | 135 | 0.04478 | 0.075756 | 0.554448 | -0.1037 | 0.193262 | 1.045798 | 0.901494 | 1.213201 |
| 68 | finn-b-I9_CORATHER | ebi-a-GCST90018929 | Thyroid cancer \|\| id:ebi-a-GCST90018929 | \|\| id:finn-b-I9_CORATHER | Inverse variance weighted | 135 | 0.053322 | 0.046389 | 0.250364 | -0.0376 | 0.144244 | 1.05477 | 0.963098 | 1.155166 |
| 69 | finn-b-I9_CORATHER | ebi-a-GCST90018929 | Thyroid cancer \|\| id:ebi-a-GCST90018929 | \|\| id:finn-b-I9_CORATHER | Simple mode | 135 | -0.22599 | 0.196546 | 0.25227 | -0.61122 | 0.15924 | 0.797725 | 0.542688 | 1.172619 |
| 70 | finn-b-I9_CORATHER | ebi-a-GCST90018929 | Thyroid cancer \|\| id:ebi-a-GCST90018929 | \|\| id:finn-b-I9_CORATHER | Weighted mode | 135 | 0.259503 | 0.119155 | 0.031166 | 0.02596 | 0.493047 | 1.296286 | 1.0263 | 1.637297 |
| 71 | finn-b-I9_CORATHER | finn-b-C3_DLBCL | Diffuse large B-cell lymphoma \|\| id:finn-b-C3_DLBCL | \|\| id:finn-b-I9_CORATHER | MR Egger | 135 | -0.04787 | 0.269067 | 0.85906 | -0.57524 | 0.479501 | 0.953257 | 0.562568 | 1.615268 |
| 72 | finn-b-I9_CORATHER | finn-b-C3_DLBCL | Diffuse large B-cell lymphoma \|\| id:finn-b-C3_DLBCL | \|\| id:finn-b-I9_CORATHER | Weighted median | 135 | -0.30392 | 0.194782 | 0.118692 | -0.68569 | 0.077857 | 0.737924 | 0.503744 | 1.080968 |
| 73 | finn-b-I9_CORATHER | finn-b-C3_DLBCL | Diffuse large B-cell lymphoma \|\| id:finn-b-C3_DLBCL | \|\| id:finn-b-I9_CORATHER | Inverse variance weighted | 135 | -0.24057 | 0.123203 | 0.050867 | -0.48205 | 0.000911 | 0.786182 | 0.617519 | 1.000912 |
| 74 | finn-b-I9_CORATHER | finn-b-C3_DLBCL | Diffuse large B-cell lymphoma \|\| id:finn-b-C3_DLBCL | \|\| id:finn-b-I9_CORATHER | Simple mode | 135 | -0.81665 | 0.506069 | 0.108941 | -1.80855 | 0.175243 | 0.441909 | 0.163892 | 1.191535 |
| 75 | finn-b-I9_CORATHER | finn-b-C3_DLBCL | Diffuse large B-cell lymphoma \|\| id:finn-b-C3_DLBCL | \|\| id:finn-b-I9_CORATHER | Weighted mode | 135 | -0.22775 | 0.374149 | 0.543739 | -0.96108 | 0.505579 | 0.796321 | 0.382478 | 1.657945 |
| 76 | finn-b-I9_CORATHER | finn-b-C3_GBM | Brain glioblastoma \|\| id:finn-b-C3_GBM | \|\| id:finn-b-I9_CORATHER | MR Egger | 135 | -0.18264 | 0.410921 | 0.657437 | -0.98804 | 0.62277 | 0.833072 | 0.372305 | 1.864084 |
| 77 | finn-b-I9_CORATHER | finn-b-C3_GBM | Brain glioblastoma \|\| id:finn-b-C3_GBM | \|\| id:finn-b-I9_CORATHER | Weighted median | 135 | -0.09742 | 0.281232 | 0.729052 | -0.64863 | 0.453799 | 0.907179 | 0.522762 | 1.574282 |
| 78 | finn-b-I9_CORATHER | finn-b-C3_GBM | Brain glioblastoma \|\| id:finn-b-C3_GBM | \|\| id:finn-b-I9_CORATHER | Inverse variance weighted | 135 | -0.22314 | 0.187598 | 0.234252 | -0.59084 | 0.144548 | 0.8 | 0.553864 | 1.155518 |
| 79 | finn-b-I9_CORATHER | finn-b-C3_GBM | Brain glioblastoma \|\| id:finn-b-C3_GBM | \|\| id:finn-b-I9_CORATHER | Simple mode | 135 | 0.479193 | 0.792216 | 0.546284 | -1.07355 | 2.031938 | 1.614771 | 0.341793 | 7.628854 |
| 80 | finn-b-I9_CORATHER | finn-b-C3_GBM | Brain glioblastoma \|\| id:finn-b-C3_GBM | \|\| id:finn-b-I9_CORATHER | Weighted mode | 135 | 0.445899 | 0.826191 | 0.590297 | -1.17344 | 2.065234 | 1.561894 | 0.309302 | 7.887145 |
| 81 | finn-b-I9_CORATHER | finn-b-C3_MESOTHELIOMA | Mesothelioma \|\| id:finn-b-C3_MESOTHELIOMA | \|\| id:finn-b-I9_CORATHER | MR Egger | 135 | 0.377612 | 0.334347 | 0.26076 | -0.27771 | 1.032932 | 1.458797 | 0.757519 | 2.80929 |
| 82 | finn-b-I9_CORATHER | finn-b-C3_MESOTHELIOMA | Mesothelioma \|\| id:finn-b-C3_MESOTHELIOMA | \|\| id:finn-b-I9_CORATHER | Weighted median | 135 | -0.01958 | 0.241666 | 0.935413 | -0.49325 | 0.454081 | 0.980607 | 0.610639 | 1.574726 |
| 83 | finn-b-I9_CORATHER | finn-b-C3_MESOTHELIOMA | Mesothelioma \|\| id:finn-b-C3_MESOTHELIOMA | \|\| id:finn-b-I9_CORATHER | Inverse variance weighted | 135 | 0.04607 | 0.153459 | 0.764017 | -0.25471 | 0.34685 | 1.047148 | 0.775142 | 1.414604 |
| 84 | finn-b-I9_CORATHER | finn-b-C3_MESOTHELIOMA | Mesothelioma \|\| id:finn-b-C3_MESOTHELIOMA | \|\| id:finn-b-I9_CORATHER | Simple mode | 135 | -0.2712 | 0.624743 | 0.664909 | -1.4957 | 0.953291 | 0.76246 | 0.224092 | 2.594233 |
| 85 | finn-b-I9_CORATHER | finn-b-C3_MESOTHELIOMA | Mesothelioma \|\| id:finn-b-C3_MESOTHELIOMA | \|\| id:finn-b-I9_CORATHER | Weighted mode | 135 | -0.49165 | 0.437158 | 0.262751 | -1.34848 | 0.365183 | 0.611619 | 0.259636 | 1.440778 |
| 86 | finn-b-I9_CORATHER | finn-b-C3_TESTIS | Malignant neoplasm of testis \|\| id:finn-b-C3_TESTIS | \|\| id:finn-b-I9_CORATHER | MR Egger | 135 | -0.00226 | 0.269325 | 0.99333 | -0.53013 | 0.525621 | 0.997747 | 0.588527 | 1.691508 |
| 87 | finn-b-I9_CORATHER | finn-b-C3_TESTIS | Malignant neoplasm of testis \|\| id:finn-b-C3_TESTIS | \|\| id:finn-b-I9_CORATHER | Weighted median | 135 | -0.01243 | 0.193037 | 0.948642 | -0.39079 | 0.365918 | 0.987643 | 0.676525 | 1.441837 |
| 88 | finn-b-I9_CORATHER | finn-b-C3_TESTIS | Malignant neoplasm of testis \|\| id:finn-b-C3_TESTIS | \|\| id:finn-b-I9_CORATHER | Inverse variance weighted | 135 | -0.14856 | 0.124008 | 0.230917 | -0.39162 | 0.094494 | 0.861947 | 0.675962 | 1.099103 |
| 89 | finn-b-I9_CORATHER | finn-b-C3_TESTIS | Malignant neoplasm of testis \|\| id:finn-b-C3_TESTIS | \|\| id:finn-b-I9_CORATHER | Simple mode | 135 | 0.030126 | 0.485065 | 0.95057 | -0.9206 | 0.980853 | 1.030584 | 0.398279 | 2.66673 |
| 90 | finn-b-I9_CORATHER | finn-b-C3_TESTIS | Malignant neoplasm of testis \|\| id:finn-b-C3_TESTIS | \|\| id:finn-b-I9_CORATHER | Weighted mode | 135 | 0.129388 | 0.371225 | 0.727979 | -0.59821 | 0.856988 | 1.138132 | 0.549793 | 2.356055 |
| 91 | finn-b-I9_CORATHER | finn-b-CD2_HODGKIN_LYMPHOMA | Hodgkin lymphoma \|\| id:finn-b-CD2_HODGKIN_LYMPHOMA | \|\| id:finn-b-I9_CORATHER | MR Egger | 135 | -0.10027 | 0.219935 | 0.649196 | -0.53134 | 0.330801 | 0.904592 | 0.587815 | 1.392083 |
| 92 | finn-b-I9_CORATHER | finn-b-CD2_HODGKIN_LYMPHOMA | Hodgkin lymphoma \|\| id:finn-b-CD2_HODGKIN_LYMPHOMA | \|\| id:finn-b-I9_CORATHER | Weighted median | 135 | -0.06773 | 0.154176 | 0.660464 | -0.36991 | 0.23446 | 0.934517 | 0.690796 | 1.264226 |
| 93 | finn-b-I9_CORATHER | finn-b-CD2_HODGKIN_LYMPHOMA | Hodgkin lymphoma \|\| id:finn-b-CD2_HODGKIN_LYMPHOMA | \|\| id:finn-b-I9_CORATHER | Inverse variance weighted | 135 | -0.0671 | 0.100681 | 0.505093 | -0.26444 | 0.130231 | 0.935098 | 0.767638 | 1.139091 |
| 94 | finn-b-I9_CORATHER | finn-b-CD2_HODGKIN_LYMPHOMA | Hodgkin lymphoma \|\| id:finn-b-CD2_HODGKIN_LYMPHOMA | \|\| id:finn-b-I9_CORATHER | Simple mode | 135 | 0.016025 | 0.386309 | 0.966974 | -0.74114 | 0.773191 | 1.016154 | 0.47657 | 2.166668 |
| 95 | finn-b-I9_CORATHER | finn-b-CD2_HODGKIN_LYMPHOMA | Hodgkin lymphoma \|\| id:finn-b-CD2_HODGKIN_LYMPHOMA | \|\| id:finn-b-I9_CORATHER | Weighted mode | 135 | 0.164515 | 0.277591 | 0.554412 | -0.37956 | 0.708593 | 1.178821 | 0.684161 | 2.031132 |
| 96 | finn-b-I9_CORATHER | finn-b-CD2_TNK_LYMPHOMA | Mature T/NK-cell lymphomas \|\| id:finn-b-CD2_TNK_LYMPHOMA | \|\| id:finn-b-I9_CORATHER | MR Egger | 135 | -0.49834 | 0.310967 | 0.111408 | -1.10783 | 0.111157 | 0.607539 | 0.330273 | 1.117571 |
| 97 | finn-b-I9_CORATHER | finn-b-CD2_TNK_LYMPHOMA | Mature T/NK-cell lymphomas \|\| id:finn-b-CD2_TNK_LYMPHOMA | \|\| id:finn-b-I9_CORATHER | Weighted median | 135 | -0.19137 | 0.233853 | 0.41317 | -0.64972 | 0.266984 | 0.825828 | 0.522191 | 1.306019 |
| 98 | finn-b-I9_CORATHER | finn-b-CD2_TNK_LYMPHOMA | Mature T/NK-cell lymphomas \|\| id:finn-b-CD2_TNK_LYMPHOMA | \|\| id:finn-b-I9_CORATHER | Inverse variance weighted | 135 | -0.00496 | 0.142664 | 0.97227 | -0.28458 | 0.274662 | 0.995053 | 0.75233 | 1.316086 |
| 99 | finn-b-I9_CORATHER | finn-b-CD2_TNK_LYMPHOMA | Mature T/NK-cell lymphomas \|\| id:finn-b-CD2_TNK_LYMPHOMA | \|\| id:finn-b-I9_CORATHER | Simple mode | 135 | -0.20445 | 0.567751 | 0.719332 | -1.31725 | 0.90834 | 0.815093 | 0.267872 | 2.480201 |
| 100 | finn-b-I9_CORATHER | finn-b-CD2_TNK_LYMPHOMA | Mature T/NK-cell lymphomas \|\| id:finn-b-CD2_TNK_LYMPHOMA | \|\| id:finn-b-I9_CORATHER | Weighted mode | 135 | -0.34081 | 0.400859 | 0.396732 | -1.12649 | 0.444875 | 0.711194 | 0.324168 | 1.560295 |
| 101 | finn-b-I9_CORATHER | ieu-b-4874 | Bladder cancer \|\| id:ieu-b-4874 | \|\| id:finn-b-I9_CORATHER | MR Egger | 126 | 0.000204 | 0.000306 | 0.506904 | -0.0004 | 0.000804 | 1.000204 | 0.999604 | 1.000805 |
| 102 | finn-b-I9_CORATHER | ieu-b-4874 | Bladder cancer \|\| id:ieu-b-4874 | \|\| id:finn-b-I9_CORATHER | Weighted median | 126 | 2.97E-05 | 0.000251 | 0.905627 | -0.00046 | 0.000521 | 1.00003 | 0.999538 | 1.000521 |
| 103 | finn-b-I9_CORATHER | ieu-b-4874 | Bladder cancer \|\| id:ieu-b-4874 | \|\| id:finn-b-I9_CORATHER | Inverse variance weighted | 126 | 0.000232 | 0.000161 | 0.151081 | ###### | 0.000548 | 1.000232 | 0.999915 | 1.000548 |
| 104 | finn-b-I9_CORATHER | ieu-b-4874 | Bladder cancer \|\| id:ieu-b-4874 | \|\| id:finn-b-I9_CORATHER | Simple mode | 126 | ###### | 0.000566 | 0.890882 | -0.00119 | 0.001031 | 0.999922 | 0.998814 | 1.001031 |
| 105 | finn-b-I9_CORATHER | ieu-b-4874 | Bladder cancer \|\| id:ieu-b-4874 | \|\| id:finn-b-I9_CORATHER | Weighted mode | 126 | ###### | 0.00036 | 0.829453 | -0.00078 | 0.000628 | 0.999922 | 0.999217 | 1.000628 |
| 106 | finn-b-I9_CORATHER | ieu-b-4912 | Head and neck cancer \|\| id:ieu-b-4912 | \|\| id:finn-b-I9_CORATHER | MR Egger | 123 | 0.000109 | 0.000281 | 0.6997 | -0.00044 | 0.000659 | 1.000109 | 0.999558 | 1.000659 |
| 107 | finn-b-I9_CORATHER | ieu-b-4912 | Head and neck cancer \|\| id:ieu-b-4912 | \|\| id:finn-b-I9_CORATHER | Weighted median | 123 | -0.00034 | 0.000232 | 0.145224 | -0.00079 | 0.000117 | 0.999662 | 0.999206 | 1.000117 |
| 108 | finn-b-I9_CORATHER | ieu-b-4912 | Head and neck cancer \|\| id:ieu-b-4912 | \|\| id:finn-b-I9_CORATHER | Inverse variance weighted | 123 | -0.00024 | 0.000148 | 0.110062 | -0.00053 | 5.34E-05 | 0.999764 | 0.999475 | 1.000053 |
| 109 | finn-b-I9_CORATHER | ieu-b-4912 | Head and neck cancer \|\| id:ieu-b-4912 | \|\| id:finn-b-I9_CORATHER | Simple mode | 123 | -0.00018 | 0.000599 | 0.759509 | -0.00136 | 0.000989 | 0.999816 | 0.998644 | 1.00099 |
| 110 | finn-b-I9_CORATHER | ieu-b-4912 | Head and neck cancer \|\| id:ieu-b-4912 | \|\| id:finn-b-I9_CORATHER | Weighted mode | 123 | -0.00065 | 0.000383 | 0.093758 | -0.0014 | 0.000104 | 0.999353 | 0.998604 | 1.000104 |
| 111 | finn-b-I9_CORATHER | ukb-a-60 | Cancer code self-reported: squamous cell carcinoma \|\| id:ukb-a-60 | \|\| id:finn-b-I9_CORATHER | MR Egger | 129 | -0.00017 | 0.000185 | 0.362065 | -0.00053 | 0.000194 | 0.999831 | 0.999468 | 1.000194 |
| 112 | finn-b-I9_CORATHER | ukb-a-60 | Cancer code self-reported: squamous cell carcinoma \|\| id:ukb-a-60 | \|\| id:finn-b-I9_CORATHER | Weighted median | 129 | 0.00011 | 0.000157 | 0.482051 | -0.0002 | 0.000417 | 1.00011 | 0.999803 | 1.000417 |
| 113 | finn-b-I9_CORATHER | ukb-a-60 | Cancer code self-reported: squamous cell carcinoma \|\| id:ukb-a-60 | \|\| id:finn-b-I9_CORATHER | Inverse variance weighted | 129 | ###### | 9.74E-05 | 0.813209 | -0.00021 | 0.000168 | 0.999977 | 0.999786 | 1.000168 |
| 114 | finn-b-I9_CORATHER | ukb-a-60 | Cancer code self-reported: squamous cell carcinoma \|\| id:ukb-a-60 | \|\| id:finn-b-I9_CORATHER | Simple mode | 129 | -0.00012 | 0.00032 | 0.704077 | -0.00075 | 0.000505 | 0.999878 | 0.999251 | 1.000506 |
| 115 | finn-b-I9_CORATHER | ukb-a-60 | Cancer code self-reported: squamous cell carcinoma \|\| id:ukb-a-60 | \|\| id:finn-b-I9_CORATHER | Weighted mode | 129 | 8.06E-05 | 0.000215 | 0.708894 | -0.00034 | 0.000503 | 1.000081 | 0.999658 | 1.000503 |

**Table S2:** Reverse Mendelian Randomization Analysis Assessing the Causality Between Coronary Atherosclerosis and 23 Types of Cancer.

|  | id.exposure | id.outcome | outcome | exposure | method | nsnp | b | se | pval | lo_ci | up_ci | or | or_lci95 | or_uci95 |
| --- | --- | --- | --- | --- | --- | --- | --- | --- | --- | --- | --- | --- | --- | --- |
| 1 | ebi-a-GCST004744 | finn-b-I9_CORATHER | Coronary atherosclerosis \|\| id:finn-b-I9_CORATHER | \|\| id:ebi-a-GCST004744 | MR Egger | 63 | -0.05768 | 0.047568 | 0.229975 | -0.15091 | 0.035554 | 0.943954 | 0.859924 | 1.036194 |
| 2 | ebi-a-GCST004744 | finn-b-I9_CORATHER | Coronary atherosclerosis \|\| id:finn-b-I9_CORATHER | \|\| id:ebi-a-GCST004744 | Weighted median | 63 | -0.01996 | 0.026034 | 0.443376 | -0.07098 | 0.031072 | 0.980242 | 0.931478 | 1.03156 |
| 3 | ebi-a-GCST004744 | finn-b-I9_CORATHER | Coronary atherosclerosis \|\| id:finn-b-I9_CORATHER | \|\| id:ebi-a-GCST004744 | Inverse variance weighted | 63 | -0.01068 | 0.019783 | 0.589173 | -0.04946 | 0.028091 | 0.989373 | 0.951746 | 1.028489 |
| 4 | ebi-a-GCST004744 | finn-b-I9_CORATHER | Coronary atherosclerosis \|\| id:finn-b-I9_CORATHER | \|\| id:ebi-a-GCST004744 | Simple mode | 63 | -0.00138 | 0.050423 | 0.97819 | -0.10021 | 0.097445 | 0.998617 | 0.904644 | 1.102351 |
| 5 | ebi-a-GCST004744 | finn-b-I9_CORATHER | Coronary atherosclerosis \|\| id:finn-b-I9_CORATHER | \|\| id:ebi-a-GCST004744 | Weighted mode | 63 | -0.01626 | 0.032192 | 0.615246 | -0.07936 | 0.046835 | 0.98387 | 0.923709 | 1.047949 |
| 6 | ebi-a-GCST90018799 | finn-b-I9_CORATHER | Coronary atherosclerosis \|\| id:finn-b-I9_CORATHER | \|\| id:ebi-a-GCST90018799 | MR Egger | 147 | 0.054849 | 0.044588 | 0.220636 | -0.03254 | 0.142241 | 1.056381 | 0.967981 | 1.152855 |
| 7 | ebi-a-GCST90018799 | finn-b-I9_CORATHER | Coronary atherosclerosis \|\| id:finn-b-I9_CORATHER | \|\| id:ebi-a-GCST90018799 | Weighted median | 147 | 0.033286 | 0.027788 | 0.230967 | -0.02118 | 0.08775 | 1.033846 | 0.979045 | 1.091715 |
| 8 | ebi-a-GCST90018799 | finn-b-I9_CORATHER | Coronary atherosclerosis \|\| id:finn-b-I9_CORATHER | \|\| id:ebi-a-GCST90018799 | Inverse variance weighted | 147 | -0.00698 | 0.019977 | 0.726952 | -0.04613 | 0.032179 | 0.993049 | 0.954917 | 1.032703 |
| 9 | ebi-a-GCST90018799 | finn-b-I9_CORATHER | Coronary atherosclerosis \|\| id:finn-b-I9_CORATHER | \|\| id:ebi-a-GCST90018799 | Simple mode | 147 | -0.04493 | 0.066233 | 0.498607 | -0.17475 | 0.084886 | 0.956064 | 0.839669 | 1.088593 |
| 10 | ebi-a-GCST90018799 | finn-b-I9_CORATHER | Coronary atherosclerosis \|\| id:finn-b-I9_CORATHER | \|\| id:ebi-a-GCST90018799 | Weighted mode | 147 | 0.037649 | 0.034044 | 0.270593 | -0.02908 | 0.104376 | 1.038367 | 0.971341 | 1.110018 |
| 11 | ebi-a-GCST90018803 | finn-b-I9_CORATHER | Coronary atherosclerosis \|\| id:finn-b-I9_CORATHER | \|\| id:ebi-a-GCST90018803 | MR Egger | 17 | -0.03901 | 0.01483 | 0.018925 | -0.06807 | -0.00994 | 0.961746 | 0.934194 | 0.99011 |
| 12 | ebi-a-GCST90018803 | finn-b-I9_CORATHER | Coronary atherosclerosis \|\| id:finn-b-I9_CORATHER | \|\| id:ebi-a-GCST90018803 | Weighted median | 17 | -0.02362 | 0.013178 | 0.073066 | -0.04945 | 0.002208 | 0.976655 | 0.951752 | 1.002211 |
| 13 | ebi-a-GCST90018803 | finn-b-I9_CORATHER | Coronary atherosclerosis \|\| id:finn-b-I9_CORATHER | \|\| id:ebi-a-GCST90018803 | Inverse variance weighted | 17 | -0.0166 | 0.010583 | 0.116824 | -0.03734 | 0.004146 | 0.98354 | 0.96335 | 1.004154 |
| 14 | ebi-a-GCST90018803 | finn-b-I9_CORATHER | Coronary atherosclerosis \|\| id:finn-b-I9_CORATHER | \|\| id:ebi-a-GCST90018803 | Simple mode | 17 | -0.02414 | 0.018114 | 0.201345 | -0.05964 | 0.011366 | 0.976151 | 0.942101 | 1.011431 |
| 15 | ebi-a-GCST90018803 | finn-b-I9_CORATHER | Coronary atherosclerosis \|\| id:finn-b-I9_CORATHER | \|\| id:ebi-a-GCST90018803 | Weighted mode | 17 | -0.01944 | 0.014504 | 0.198935 | -0.04786 | 0.008991 | 0.980751 | 0.953263 | 1.009032 |
| 16 | ebi-a-GCST90018808 | finn-b-I9_CORATHER | Coronary atherosclerosis \|\| id:finn-b-I9_CORATHER | \|\| id:ebi-a-GCST90018808 | MR Egger | 107 | 0.05794 | 0.067747 | 0.394366 | -0.07484 | 0.190724 | 1.059651 | 0.927888 | 1.210125 |
| 17 | ebi-a-GCST90018808 | finn-b-I9_CORATHER | Coronary atherosclerosis \|\| id:finn-b-I9_CORATHER | \|\| id:ebi-a-GCST90018808 | Weighted median | 107 | -0.00647 | 0.026661 | 0.808183 | -0.05873 | 0.045782 | 0.993549 | 0.942964 | 1.046846 |
| 18 | ebi-a-GCST90018808 | finn-b-I9_CORATHER | Coronary atherosclerosis \|\| id:finn-b-I9_CORATHER | \|\| id:ebi-a-GCST90018808 | Inverse variance weighted | 107 | -0.05144 | 0.03242 | 0.112556 | -0.11499 | 0.012099 | 0.949857 | 0.891378 | 1.012172 |
| 19 | ebi-a-GCST90018808 | finn-b-I9_CORATHER | Coronary atherosclerosis \|\| id:finn-b-I9_CORATHER | \|\| id:ebi-a-GCST90018808 | Simple mode | 107 | -0.00019 | 0.045613 | 0.996611 | -0.0896 | 0.089207 | 0.999806 | 0.914301 | 1.093307 |
| 20 | ebi-a-GCST90018808 | finn-b-I9_CORATHER | Coronary atherosclerosis \|\| id:finn-b-I9_CORATHER | \|\| id:ebi-a-GCST90018808 | Weighted mode | 107 | -0.00019 | 0.031808 | 0.995141 | -0.06254 | 0.06215 | 0.999806 | 0.939377 | 1.064122 |
| 21 | ebi-a-GCST90018817 | finn-b-I9_CORATHER | Coronary atherosclerosis \|\| id:finn-b-I9_CORATHER | \|\| id:ebi-a-GCST90018817 | MR Egger | 40 | -0.00472 | 0.029688 | 0.874581 | -0.06291 | 0.053471 | 0.995293 | 0.939031 | 1.054927 |
| 22 | ebi-a-GCST90018817 | finn-b-I9_CORATHER | Coronary atherosclerosis \|\| id:finn-b-I9_CORATHER | \|\| id:ebi-a-GCST90018817 | Weighted median | 40 | 0.000966 | 0.016733 | 0.953945 | -0.03183 | 0.033763 | 1.000967 | 0.968671 | 1.034339 |
| 23 | ebi-a-GCST90018817 | finn-b-I9_CORATHER | Coronary atherosclerosis \|\| id:finn-b-I9_CORATHER | \|\| id:ebi-a-GCST90018817 | Inverse variance weighted | 40 | -0.00666 | 0.013367 | 0.618329 | -0.03286 | 0.019539 | 0.993363 | 0.967676 | 1.019731 |
| 24 | ebi-a-GCST90018817 | finn-b-I9_CORATHER | Coronary atherosclerosis \|\| id:finn-b-I9_CORATHER | \|\| id:ebi-a-GCST90018817 | Simple mode | 40 | -0.00577 | 0.026648 | 0.829818 | -0.058 | 0.046464 | 0.99425 | 0.943653 | 1.047561 |
| 25 | ebi-a-GCST90018817 | finn-b-I9_CORATHER | Coronary atherosclerosis \|\| id:finn-b-I9_CORATHER | \|\| id:ebi-a-GCST90018817 | Weighted mode | 40 | 0.014374 | 0.026082 | 0.584693 | -0.03675 | 0.065494 | 1.014478 | 0.963921 | 1.067687 |
| 26 | ebi-a-GCST90018841 | finn-b-I9_CORATHER | Coronary atherosclerosis \|\| id:finn-b-I9_CORATHER | \|\| id:ebi-a-GCST90018841 | MR Egger | 44 | 0.00491 | 0.014475 | 0.736138 | -0.02346 | 0.03328 | 1.004922 | 0.976813 | 1.03384 |
| 27 | ebi-a-GCST90018841 | finn-b-I9_CORATHER | Coronary atherosclerosis \|\| id:finn-b-I9_CORATHER | \|\| id:ebi-a-GCST90018841 | Weighted median | 44 | 0.018567 | 0.014874 | 0.211919 | -0.01059 | 0.04772 | 1.018741 | 0.98947 | 1.048877 |
| 28 | ebi-a-GCST90018841 | finn-b-I9_CORATHER | Coronary atherosclerosis \|\| id:finn-b-I9_CORATHER | \|\| id:ebi-a-GCST90018841 | Inverse variance weighted | 44 | 0.005928 | 0.010305 | 0.56513 | -0.01427 | 0.026125 | 1.005945 | 0.985832 | 1.026469 |
| 29 | ebi-a-GCST90018841 | finn-b-I9_CORATHER | Coronary atherosclerosis \|\| id:finn-b-I9_CORATHER | \|\| id:ebi-a-GCST90018841 | Simple mode | 44 | -0.01428 | 0.026766 | 0.59631 | -0.06675 | 0.038177 | 0.985817 | 0.935433 | 1.038915 |
| 30 | ebi-a-GCST90018841 | finn-b-I9_CORATHER | Coronary atherosclerosis \|\| id:finn-b-I9_CORATHER | \|\| id:ebi-a-GCST90018841 | Weighted mode | 44 | 0.014509 | 0.013385 | 0.28443 | -0.01173 | 0.040744 | 1.014615 | 0.988342 | 1.041585 |
| 31 | ebi-a-GCST90018849 | finn-b-I9_CORATHER | Coronary atherosclerosis \|\| id:finn-b-I9_CORATHER | \|\| id:ebi-a-GCST90018849 | MR Egger | 49 | -0.00688 | 0.016611 | 0.680532 | -0.03944 | 0.025675 | 0.993142 | 0.961328 | 1.026008 |
| 32 | ebi-a-GCST90018849 | finn-b-I9_CORATHER | Coronary atherosclerosis \|\| id:finn-b-I9_CORATHER | \|\| id:ebi-a-GCST90018849 | Weighted median | 49 | -0.02238 | 0.01611 | 0.16474 | -0.05396 | 0.009194 | 0.977868 | 0.947474 | 1.009236 |
| 33 | ebi-a-GCST90018849 | finn-b-I9_CORATHER | Coronary atherosclerosis \|\| id:finn-b-I9_CORATHER | \|\| id:ebi-a-GCST90018849 | Inverse variance weighted | 49 | -0.01382 | 0.012668 | 0.275369 | -0.03865 | 0.011012 | 0.986277 | 0.962089 | 1.011072 |
| 34 | ebi-a-GCST90018849 | finn-b-I9_CORATHER | Coronary atherosclerosis \|\| id:finn-b-I9_CORATHER | \|\| id:ebi-a-GCST90018849 | Simple mode | 49 | 0.001131 | 0.024871 | 0.963924 | -0.04762 | 0.049878 | 1.001131 | 0.9535 | 1.051143 |
| 35 | ebi-a-GCST90018849 | finn-b-I9_CORATHER | Coronary atherosclerosis \|\| id:finn-b-I9_CORATHER | \|\| id:ebi-a-GCST90018849 | Weighted mode | 49 | -0.01607 | 0.013534 | 0.240904 | -0.0426 | 0.010456 | 0.984058 | 0.958298 | 1.010511 |
| 36 | ebi-a-GCST90018858 | finn-b-I9_CORATHER | Coronary atherosclerosis \|\| id:finn-b-I9_CORATHER | \|\| id:ebi-a-GCST90018858 | MR Egger | 46 | -0.01395 | 0.009497 | 0.149064 | -0.03256 | 0.004667 | 0.98615 | 0.967964 | 1.004678 |
| 37 | ebi-a-GCST90018858 | finn-b-I9_CORATHER | Coronary atherosclerosis \|\| id:finn-b-I9_CORATHER | \|\| id:ebi-a-GCST90018858 | Weighted median | 46 | -0.00818 | 0.00943 | 0.385747 | -0.02666 | 0.010304 | 0.991854 | 0.97369 | 1.010357 |
| 38 | ebi-a-GCST90018858 | finn-b-I9_CORATHER | Coronary atherosclerosis \|\| id:finn-b-I9_CORATHER | \|\| id:ebi-a-GCST90018858 | Inverse variance weighted | 46 | -0.01036 | 0.006931 | 0.134827 | -0.02395 | 0.00322 | 0.98969 | 0.976336 | 1.003226 |
| 39 | ebi-a-GCST90018858 | finn-b-I9_CORATHER | Coronary atherosclerosis \|\| id:finn-b-I9_CORATHER | \|\| id:ebi-a-GCST90018858 | Simple mode | 46 | 0.026197 | 0.01514 | 0.090437 | -0.00348 | 0.055872 | 1.026543 | 0.996528 | 1.057462 |
| 40 | ebi-a-GCST90018858 | finn-b-I9_CORATHER | Coronary atherosclerosis \|\| id:finn-b-I9_CORATHER | \|\| id:ebi-a-GCST90018858 | Weighted mode | 46 | -0.01664 | 0.010372 | 0.115672 | -0.03697 | 0.003691 | 0.983499 | 0.963706 | 1.003698 |
| 41 | ebi-a-GCST90018875 | finn-b-I9_CORATHER | Coronary atherosclerosis \|\| id:finn-b-I9_CORATHER | \|\| id:ebi-a-GCST90018875 | MR Egger | 39 | 0.002695 | 0.052036 | 0.958976 | -0.09929 | 0.104685 | 1.002698 | 0.905476 | 1.11036 |
| 42 | ebi-a-GCST90018875 | finn-b-I9_CORATHER | Coronary atherosclerosis \|\| id:finn-b-I9_CORATHER | \|\| id:ebi-a-GCST90018875 | Weighted median | 39 | -0.03158 | 0.033846 | 0.350792 | -0.09792 | 0.034758 | 0.968914 | 0.906724 | 1.035369 |
| 43 | ebi-a-GCST90018875 | finn-b-I9_CORATHER | Coronary atherosclerosis \|\| id:finn-b-I9_CORATHER | \|\| id:ebi-a-GCST90018875 | Inverse variance weighted | 39 | 0.015088 | 0.023508 | 0.521 | -0.03099 | 0.061163 | 1.015202 | 0.969487 | 1.063072 |
| 44 | ebi-a-GCST90018875 | finn-b-I9_CORATHER | Coronary atherosclerosis \|\| id:finn-b-I9_CORATHER | \|\| id:ebi-a-GCST90018875 | Simple mode | 39 | -0.05436 | 0.057999 | 0.354518 | -0.16804 | 0.059315 | 0.947088 | 0.845318 | 1.06111 |
| 45 | ebi-a-GCST90018875 | finn-b-I9_CORATHER | Coronary atherosclerosis \|\| id:finn-b-I9_CORATHER | \|\| id:ebi-a-GCST90018875 | Weighted mode | 39 | -0.04755 | 0.050557 | 0.352906 | -0.14664 | 0.051543 | 0.953565 | 0.863606 | 1.052894 |
| 46 | ebi-a-GCST90018888 | finn-b-I9_CORATHER | Coronary atherosclerosis \|\| id:finn-b-I9_CORATHER | \|\| id:ebi-a-GCST90018888 | MR Egger | 30 | -0.01977 | 0.026382 | 0.459893 | -0.07148 | 0.031939 | 0.980425 | 0.931016 | 1.032455 |
| 47 | ebi-a-GCST90018888 | finn-b-I9_CORATHER | Coronary atherosclerosis \|\| id:finn-b-I9_CORATHER | \|\| id:ebi-a-GCST90018888 | Weighted median | 30 | -0.02523 | 0.020439 | 0.217112 | -0.06529 | 0.014834 | 0.975088 | 0.936797 | 1.014945 |
| 48 | ebi-a-GCST90018888 | finn-b-I9_CORATHER | Coronary atherosclerosis \|\| id:finn-b-I9_CORATHER | \|\| id:ebi-a-GCST90018888 | Inverse variance weighted | 30 | -0.03325 | 0.014184 | 0.019079 | -0.06105 | -0.00545 | 0.967299 | 0.940777 | 0.994568 |
| 49 | ebi-a-GCST90018888 | finn-b-I9_CORATHER | Coronary atherosclerosis \|\| id:finn-b-I9_CORATHER | \|\| id:ebi-a-GCST90018888 | Simple mode | 30 | -0.01913 | 0.036083 | 0.600128 | -0.08985 | 0.051598 | 0.981057 | 0.91407 | 1.052952 |
| 50 | ebi-a-GCST90018888 | finn-b-I9_CORATHER | Coronary atherosclerosis \|\| id:finn-b-I9_CORATHER | \|\| id:ebi-a-GCST90018888 | Weighted mode | 30 | -0.03201 | 0.030661 | 0.305065 | -0.09211 | 0.028083 | 0.968494 | 0.912005 | 1.028481 |
| 51 | ebi-a-GCST90018893 | finn-b-I9_CORATHER | Coronary atherosclerosis \|\| id:finn-b-I9_CORATHER | \|\| id:ebi-a-GCST90018893 | MR Egger | 40 | -0.00929 | 0.021167 | 0.663371 | -0.05077 | 0.032202 | 0.990757 | 0.950494 | 1.032726 |
| 52 | ebi-a-GCST90018893 | finn-b-I9_CORATHER | Coronary atherosclerosis \|\| id:finn-b-I9_CORATHER | \|\| id:ebi-a-GCST90018893 | Weighted median | 40 | -0.01023 | 0.015949 | 0.521149 | -0.04149 | 0.021028 | 0.98982 | 0.959356 | 1.021251 |
| 53 | ebi-a-GCST90018893 | finn-b-I9_CORATHER | Coronary atherosclerosis \|\| id:finn-b-I9_CORATHER | \|\| id:ebi-a-GCST90018893 | Inverse variance weighted | 40 | 0.006589 | 0.010545 | 0.532088 | -0.01408 | 0.027258 | 1.006611 | 0.986019 | 1.027633 |
| 54 | ebi-a-GCST90018893 | finn-b-I9_CORATHER | Coronary atherosclerosis \|\| id:finn-b-I9_CORATHER | \|\| id:ebi-a-GCST90018893 | Simple mode | 40 | 0.046572 | 0.033258 | 0.169323 | -0.01861 | 0.111758 | 1.047674 | 0.981558 | 1.118243 |
| 55 | ebi-a-GCST90018893 | finn-b-I9_CORATHER | Coronary atherosclerosis \|\| id:finn-b-I9_CORATHER | \|\| id:ebi-a-GCST90018893 | Weighted mode | 40 | -0.03348 | 0.027442 | 0.229796 | -0.08726 | 0.020307 | 0.967075 | 0.916434 | 1.020515 |
| 56 | ebi-a-GCST90018905 | finn-b-I9_CORATHER | Coronary atherosclerosis \|\| id:finn-b-I9_CORATHER | \|\| id:ebi-a-GCST90018905 | MR Egger | 173 | 0.021013 | 0.023187 | 0.366085 | -0.02443 | 0.06646 | 1.021235 | 0.975862 | 1.068718 |
| 57 | ebi-a-GCST90018905 | finn-b-I9_CORATHER | Coronary atherosclerosis \|\| id:finn-b-I9_CORATHER | \|\| id:ebi-a-GCST90018905 | Weighted median | 173 | -0.00042 | 0.017852 | 0.981448 | -0.0354 | 0.034575 | 0.999585 | 0.965214 | 1.035179 |
| 58 | ebi-a-GCST90018905 | finn-b-I9_CORATHER | Coronary atherosclerosis \|\| id:finn-b-I9_CORATHER | \|\| id:ebi-a-GCST90018905 | Inverse variance weighted | 173 | -0.00181 | 0.012038 | 0.880252 | -0.02541 | 0.021781 | 0.998188 | 0.974912 | 1.02202 |
| 59 | ebi-a-GCST90018905 | finn-b-I9_CORATHER | Coronary atherosclerosis \|\| id:finn-b-I9_CORATHER | \|\| id:ebi-a-GCST90018905 | Simple mode | 173 | -0.01165 | 0.036738 | 0.751585 | -0.08366 | 0.060359 | 0.988419 | 0.919748 | 1.062218 |
| 60 | ebi-a-GCST90018905 | finn-b-I9_CORATHER | Coronary atherosclerosis \|\| id:finn-b-I9_CORATHER | \|\| id:ebi-a-GCST90018905 | Weighted mode | 173 | -0.00032 | 0.02092 | 0.987791 | -0.04132 | 0.040682 | 0.999679 | 0.959519 | 1.041521 |
| 61 | ebi-a-GCST90018921 | finn-b-I9_CORATHER | Coronary atherosclerosis \|\| id:finn-b-I9_CORATHER | \|\| id:ebi-a-GCST90018921 | MR Egger | 149 | -0.00662 | 0.031433 | 0.833497 | -0.06823 | 0.054989 | 0.993402 | 0.934048 | 1.056529 |
| 62 | ebi-a-GCST90018921 | finn-b-I9_CORATHER | Coronary atherosclerosis \|\| id:finn-b-I9_CORATHER | \|\| id:ebi-a-GCST90018921 | Weighted median | 149 | -0.00719 | 0.024315 | 0.767524 | -0.05484 | 0.040469 | 0.992838 | 0.946632 | 1.041299 |
| 63 | ebi-a-GCST90018921 | finn-b-I9_CORATHER | Coronary atherosclerosis \|\| id:finn-b-I9_CORATHER | \|\| id:ebi-a-GCST90018921 | Inverse variance weighted | 149 | -0.02566 | 0.018791 | 0.172148 | -0.06249 | 0.011175 | 0.97467 | 0.939425 | 1.011237 |
| 64 | ebi-a-GCST90018921 | finn-b-I9_CORATHER | Coronary atherosclerosis \|\| id:finn-b-I9_CORATHER | \|\| id:ebi-a-GCST90018921 | Simple mode | 149 | -0.0158 | 0.057892 | 0.785278 | -0.12927 | 0.097667 | 0.984323 | 0.878737 | 1.102596 |
| 65 | ebi-a-GCST90018921 | finn-b-I9_CORATHER | Coronary atherosclerosis \|\| id:finn-b-I9_CORATHER | \|\| id:ebi-a-GCST90018921 | Weighted mode | 149 | 0.005077 | 0.025271 | 0.84104 | -0.04445 | 0.054609 | 1.00509 | 0.956519 | 1.056128 |
| 66 | ebi-a-GCST90018929 | finn-b-I9_CORATHER | Coronary atherosclerosis \|\| id:finn-b-I9_CORATHER | \|\| id:ebi-a-GCST90018929 | MR Egger | 25 | -0.01798 | 0.019806 | 0.37338 | -0.0568 | 0.020839 | 0.98218 | 0.944783 | 1.021058 |
| 67 | ebi-a-GCST90018929 | finn-b-I9_CORATHER | Coronary atherosclerosis \|\| id:finn-b-I9_CORATHER | \|\| id:ebi-a-GCST90018929 | Weighted median | 25 | -0.01727 | 0.015068 | 0.251882 | -0.0468 | 0.012269 | 0.982883 | 0.954279 | 1.012344 |
| 68 | ebi-a-GCST90018929 | finn-b-I9_CORATHER | Coronary atherosclerosis \|\| id:finn-b-I9_CORATHER | \|\| id:ebi-a-GCST90018929 | Inverse variance weighted | 25 | -0.01374 | 0.010297 | 0.181938 | -0.03393 | 0.006438 | 0.986349 | 0.966642 | 1.006458 |
| 69 | ebi-a-GCST90018929 | finn-b-I9_CORATHER | Coronary atherosclerosis \|\| id:finn-b-I9_CORATHER | \|\| id:ebi-a-GCST90018929 | Simple mode | 25 | -0.03557 | 0.027245 | 0.204093 | -0.08897 | 0.017831 | 0.965056 | 0.914874 | 1.017991 |
| 70 | ebi-a-GCST90018929 | finn-b-I9_CORATHER | Coronary atherosclerosis \|\| id:finn-b-I9_CORATHER | \|\| id:ebi-a-GCST90018929 | Weighted mode | 25 | -0.02843 | 0.019175 | 0.15116 | -0.06601 | 0.009152 | 0.971969 | 0.936117 | 1.009194 |
| 71 | finn-b-C3_DLBCL | finn-b-I9_CORATHER | Coronary atherosclerosis \|\| id:finn-b-I9_CORATHER | \|\| id:finn-b-C3_DLBCL | MR Egger | 16 | -0.00597 | 0.014367 | 0.684104 | -0.03413 | 0.022191 | 0.994049 | 0.966447 | 1.022439 |
| 72 | finn-b-C3_DLBCL | finn-b-I9_CORATHER | Coronary atherosclerosis \|\| id:finn-b-I9_CORATHER | \|\| id:finn-b-C3_DLBCL | Weighted median | 16 | -0.0043 | 0.00858 | 0.616125 | -0.02112 | 0.012515 | 0.995708 | 0.979104 | 1.012594 |
| 73 | finn-b-C3_DLBCL | finn-b-I9_CORATHER | Coronary atherosclerosis \|\| id:finn-b-I9_CORATHER | \|\| id:finn-b-C3_DLBCL | Inverse variance weighted | 16 | -0.00077 | 0.00643 | 0.904146 | -0.01338 | 0.011829 | 0.999226 | 0.986711 | 1.011899 |
| 74 | finn-b-C3_DLBCL | finn-b-I9_CORATHER | Coronary atherosclerosis \|\| id:finn-b-I9_CORATHER | \|\| id:finn-b-C3_DLBCL | Simple mode | 16 | -0.00557 | 0.014029 | 0.696813 | -0.03307 | 0.021925 | 0.994443 | 0.96747 | 1.022167 |
| 75 | finn-b-C3_DLBCL | finn-b-I9_CORATHER | Coronary atherosclerosis \|\| id:finn-b-I9_CORATHER | \|\| id:finn-b-C3_DLBCL | Weighted mode | 16 | -0.00597 | 0.014129 | 0.678518 | -0.03366 | 0.02172 | 0.994046 | 0.966896 | 1.021958 |
| 76 | finn-b-C3_GBM | finn-b-I9_CORATHER | Coronary atherosclerosis \|\| id:finn-b-I9_CORATHER | \|\| id:finn-b-C3_GBM | MR Egger | 16 | -0.00288 | 0.007465 | 0.705254 | -0.01751 | 0.011749 | 0.997122 | 0.98264 | 1.011818 |
| 77 | finn-b-C3_GBM | finn-b-I9_CORATHER | Coronary atherosclerosis \|\| id:finn-b-I9_CORATHER | \|\| id:finn-b-C3_GBM | Weighted median | 16 | 0.000921 | 0.005576 | 0.868828 | -0.01001 | 0.01185 | 1.000921 | 0.990041 | 1.011921 |
| 78 | finn-b-C3_GBM | finn-b-I9_CORATHER | Coronary atherosclerosis \|\| id:finn-b-I9_CORATHER | \|\| id:finn-b-C3_GBM | Inverse variance weighted | 16 | -0.00049 | 0.003926 | 0.900424 | -0.00819 | 0.007204 | 0.999509 | 0.991847 | 1.00723 |
| 79 | finn-b-C3_GBM | finn-b-I9_CORATHER | Coronary atherosclerosis \|\| id:finn-b-I9_CORATHER | \|\| id:finn-b-C3_GBM | Simple mode | 16 | -0.00108 | 0.009452 | 0.910842 | -0.0196 | 0.01745 | 0.998924 | 0.980588 | 1.017603 |
| 80 | finn-b-C3_GBM | finn-b-I9_CORATHER | Coronary atherosclerosis \|\| id:finn-b-I9_CORATHER | \|\| id:finn-b-C3_GBM | Weighted mode | 16 | 0.000389 | 0.00883 | 0.96544 | -0.01692 | 0.017696 | 1.000389 | 0.983224 | 1.017854 |
| 81 | finn-b-C3_MESOTHELIOMA | finn-b-I9_CORATHER | Coronary atherosclerosis \|\| id:finn-b-I9_CORATHER | \|\| id:finn-b-C3_MESOTHELIOMA | MR Egger | 15 | 0.007171 | 0.011753 | 0.552259 | -0.01586 | 0.030207 | 1.007197 | 0.984261 | 1.030668 |
| 82 | finn-b-C3_MESOTHELIOMA | finn-b-I9_CORATHER | Coronary atherosclerosis \|\| id:finn-b-I9_CORATHER | \|\| id:finn-b-C3_MESOTHELIOMA | Weighted median | 15 | 0.003187 | 0.007247 | 0.660097 | -0.01102 | 0.01739 | 1.003192 | 0.989044 | 1.017542 |
| 83 | finn-b-C3_MESOTHELIOMA | finn-b-I9_CORATHER | Coronary atherosclerosis \|\| id:finn-b-I9_CORATHER | \|\| id:finn-b-C3_MESOTHELIOMA | Inverse variance weighted | 15 | -0.00012 | 0.005876 | 0.983273 | -0.01164 | 0.011393 | 0.999877 | 0.988428 | 1.011458 |
| 84 | finn-b-C3_MESOTHELIOMA | finn-b-I9_CORATHER | Coronary atherosclerosis \|\| id:finn-b-I9_CORATHER | \|\| id:finn-b-C3_MESOTHELIOMA | Simple mode | 15 | 0.011823 | 0.013442 | 0.393917 | -0.01452 | 0.038169 | 1.011894 | 0.985583 | 1.038907 |
| 85 | finn-b-C3_MESOTHELIOMA | finn-b-I9_CORATHER | Coronary atherosclerosis \|\| id:finn-b-I9_CORATHER | \|\| id:finn-b-C3_MESOTHELIOMA | Weighted mode | 15 | 0.012109 | 0.013042 | 0.368912 | -0.01345 | 0.037672 | 1.012183 | 0.986636 | 1.038391 |
| 86 | finn-b-C3_TESTIS | finn-b-I9_CORATHER | Coronary atherosclerosis \|\| id:finn-b-I9_CORATHER | \|\| id:finn-b-C3_TESTIS | MR Egger | 23 | -0.01908 | 0.011156 | 0.101942 | -0.04095 | 0.002786 | 0.9811 | 0.95988 | 1.002789 |
| 87 | finn-b-C3_TESTIS | finn-b-I9_CORATHER | Coronary atherosclerosis \|\| id:finn-b-I9_CORATHER | \|\| id:finn-b-C3_TESTIS | Weighted median | 23 | -0.00437 | 0.007112 | 0.538626 | -0.01831 | 0.009566 | 0.995637 | 0.981855 | 1.009612 |
| 88 | finn-b-C3_TESTIS | finn-b-I9_CORATHER | Coronary atherosclerosis \|\| id:finn-b-I9_CORATHER | \|\| id:finn-b-C3_TESTIS | Inverse variance weighted | 23 | -0.00908 | 0.005283 | 0.085549 | -0.01944 | 0.001271 | 0.990957 | 0.980749 | 1.001272 |
| 89 | finn-b-C3_TESTIS | finn-b-I9_CORATHER | Coronary atherosclerosis \|\| id:finn-b-I9_CORATHER | \|\| id:finn-b-C3_TESTIS | Simple mode | 23 | -0.0014 | 0.013261 | 0.916862 | -0.02739 | 0.024591 | 0.998601 | 0.97298 | 1.024896 |
| 90 | finn-b-C3_TESTIS | finn-b-I9_CORATHER | Coronary atherosclerosis \|\| id:finn-b-I9_CORATHER | \|\| id:finn-b-C3_TESTIS | Weighted mode | 23 | -0.00164 | 0.01356 | 0.905086 | -0.02821 | 0.024941 | 0.998366 | 0.972182 | 1.025255 |
| 91 | finn-b-CD2_HODGKIN_LYMPHOMA | finn-b-I9_CORATHER | Coronary atherosclerosis \|\| id:finn-b-I9_CORATHER | \|\| id:finn-b-CD2_HODGKIN_LYMPHOMA | MR Egger | 23 | 0.023865 | 0.014612 | 0.117331 | -0.00478 | 0.052504 | 1.024152 | 0.995236 | 1.053907 |
| 92 | finn-b-CD2_HODGKIN_LYMPHOMA | finn-b-I9_CORATHER | Coronary atherosclerosis \|\| id:finn-b-I9_CORATHER | \|\| id:finn-b-CD2_HODGKIN_LYMPHOMA | Weighted median | 23 | 0.007818 | 0.010894 | 0.472985 | -0.01353 | 0.029169 | 1.007848 | 0.986557 | 1.029599 |
| 93 | finn-b-CD2_HODGKIN_LYMPHOMA | finn-b-I9_CORATHER | Coronary atherosclerosis \|\| id:finn-b-I9_CORATHER | \|\| id:finn-b-CD2_HODGKIN_LYMPHOMA | Inverse variance weighted | 23 | 0.014116 | 0.008152 | 0.083362 | -0.00186 | 0.030094 | 1.014216 | 0.998139 | 1.030551 |
| 94 | finn-b-CD2_HODGKIN_LYMPHOMA | finn-b-I9_CORATHER | Coronary atherosclerosis \|\| id:finn-b-I9_CORATHER | \|\| id:finn-b-CD2_HODGKIN_LYMPHOMA | Simple mode | 23 | 0.007736 | 0.020605 | 0.710923 | -0.03265 | 0.048122 | 1.007766 | 0.967878 | 1.049298 |
| 95 | finn-b-CD2_HODGKIN_LYMPHOMA | finn-b-I9_CORATHER | Coronary atherosclerosis \|\| id:finn-b-I9_CORATHER | \|\| id:finn-b-CD2_HODGKIN_LYMPHOMA | Weighted mode | 23 | 0.006701 | 0.021079 | 0.753543 | -0.03461 | 0.048015 | 1.006724 | 0.965979 | 1.049187 |
| 96 | finn-b-CD2_TNK_LYMPHOMA | finn-b-I9_CORATHER | Coronary atherosclerosis \|\| id:finn-b-I9_CORATHER | \|\| id:finn-b-CD2_TNK_LYMPHOMA | MR Egger | 21 | 0.005428 | 0.00666 | 0.425147 | -0.00763 | 0.018481 | 1.005443 | 0.992404 | 1.018653 |
| 97 | finn-b-CD2_TNK_LYMPHOMA | finn-b-I9_CORATHER | Coronary atherosclerosis \|\| id:finn-b-I9_CORATHER | \|\| id:finn-b-CD2_TNK_LYMPHOMA | Weighted median | 21 | 0.005857 | 0.006138 | 0.339969 | -0.00617 | 0.017888 | 1.005874 | 0.993846 | 1.018048 |
| 98 | finn-b-CD2_TNK_LYMPHOMA | finn-b-I9_CORATHER | Coronary atherosclerosis \|\| id:finn-b-I9_CORATHER | \|\| id:finn-b-CD2_TNK_LYMPHOMA | Inverse variance weighted | 21 | 0.004988 | 0.00432 | 0.248309 | -0.00348 | 0.013455 | 1.005 | 0.996526 | 1.013546 |
| 99 | finn-b-CD2_TNK_LYMPHOMA | finn-b-I9_CORATHER | Coronary atherosclerosis \|\| id:finn-b-I9_CORATHER | \|\| id:finn-b-CD2_TNK_LYMPHOMA | Simple mode | 21 | 0.006979 | 0.01281 | 0.591892 | -0.01813 | 0.032086 | 1.007004 | 0.982035 | 1.032607 |
| 100 | finn-b-CD2_TNK_LYMPHOMA | finn-b-I9_CORATHER | Coronary atherosclerosis \|\| id:finn-b-I9_CORATHER | \|\| id:finn-b-CD2_TNK_LYMPHOMA | Weighted mode | 21 | 0.005972 | 0.012386 | 0.634946 | -0.01831 | 0.030249 | 1.00599 | 0.981861 | 1.030712 |
| 101 | ieu-b-4874 | finn-b-I9_CORATHER | Coronary atherosclerosis \|\| id:finn-b-I9_CORATHER | \|\| id:ieu-b-4874 | MR Egger | 38 | -3.38596 | 6.098155 | 0.582162 | -15.3383 | 8.566423 | 0.033845 | 2.18E-07 | 5252.306 |
| 102 | ieu-b-4874 | finn-b-I9_CORATHER | Coronary atherosclerosis \|\| id:finn-b-I9_CORATHER | \|\| id:ieu-b-4874 | Weighted median | 38 | 1.547606 | 4.578297 | 0.73534 | -7.42585 | 10.52107 | 4.700206 | 0.000596 | 37088.7 |
| 103 | ieu-b-4874 | finn-b-I9_CORATHER | Coronary atherosclerosis \|\| id:finn-b-I9_CORATHER | \|\| id:ieu-b-4874 | Inverse variance weighted | 38 | -0.70711 | 3.10896 | 0.820079 | -6.80068 | 5.386447 | 0.493065 | 0.001113 | 218.426 |
| 104 | ieu-b-4874 | finn-b-I9_CORATHER | Coronary atherosclerosis \|\| id:finn-b-I9_CORATHER | \|\| id:ieu-b-4874 | Simple mode | 38 | 0.604916 | 7.96667 | 0.939883 | -15.0098 | 16.21959 | 1.831098 | 3.03E-07 | 11068230 |
| 105 | ieu-b-4874 | finn-b-I9_CORATHER | Coronary atherosclerosis \|\| id:finn-b-I9_CORATHER | \|\| id:ieu-b-4874 | Weighted mode | 38 | 2.811429 | 6.826953 | 0.682852 | -10.5694 | 16.19226 | 16.63366 | 2.57E-05 | 10769808 |
| 106 | ieu-b-4912 | finn-b-I9_CORATHER | Coronary atherosclerosis \|\| id:finn-b-I9_CORATHER | \|\| id:ieu-b-4912 | MR Egger | 19 | 5.652737 | 13.718 | 0.68544 | -21.2345 | 32.54001 | 285.0705 | 6.00E-10 | 1.36E+14 |
| 107 | ieu-b-4912 | finn-b-I9_CORATHER | Coronary atherosclerosis \|\| id:finn-b-I9_CORATHER | \|\| id:ieu-b-4912 | Weighted median | 19 | 7.079457 | 7.965264 | 0.374115 | -8.53246 | 22.69137 | 1187.323 | 0.000197 | 7.16E+09 |
| 108 | ieu-b-4912 | finn-b-I9_CORATHER | Coronary atherosclerosis \|\| id:finn-b-I9_CORATHER | \|\| id:ieu-b-4912 | Inverse variance weighted | 19 | 10.87567 | 6.141248 | 0.076574 | -1.16117 | 22.91252 | 52874.42 | 0.313119 | 8.93E+09 |
| 109 | ieu-b-4912 | finn-b-I9_CORATHER | Coronary atherosclerosis \|\| id:finn-b-I9_CORATHER | \|\| id:ieu-b-4912 | Simple mode | 19 | 12.99172 | 14.28038 | 0.37497 | -14.9978 | 40.98126 | 438766.3 | 3.07E-07 | 6.28E+17 |
| 110 | ieu-b-4912 | finn-b-I9_CORATHER | Coronary atherosclerosis \|\| id:finn-b-I9_CORATHER | \|\| id:ieu-b-4912 | Weighted mode | 19 | 3.290603 | 12.05393 | 0.787968 | -20.3351 | 26.9163 | 26.85906 | 1.47E-09 | 4.89E+11 |
| 111 | ukb-a-60 | finn-b-I9_CORATHER | Coronary atherosclerosis \|\| id:finn-b-I9_CORATHER | \|\| id:ukb-a-60 | MR Egger | 71 | -6.8 | 5.532996 | 0.22325 | -17.6447 | 4.044674 | 0.001114 | 2.17E-08 | 57.09257 |
| 112 | ukb-a-60 | finn-b-I9_CORATHER | Coronary atherosclerosis \|\| id:finn-b-I9_CORATHER | \|\| id:ukb-a-60 | Weighted median | 71 | -6.60765 | 4.833019 | 0.171566 | -16.0804 | 2.865071 | 0.00135 | 1.04E-07 | 17.5503 |
| 113 | ukb-a-60 | finn-b-I9_CORATHER | Coronary atherosclerosis \|\| id:finn-b-I9_CORATHER | \|\| id:ukb-a-60 | Inverse variance weighted | 71 | -0.09467 | 3.561776 | 0.978796 | -7.07575 | 6.886414 | 0.909676 | 0.000845 | 978.8846 |
| 114 | ukb-a-60 | finn-b-I9_CORATHER | Coronary atherosclerosis \|\| id:finn-b-I9_CORATHER | \|\| id:ukb-a-60 | Simple mode | 71 | -10.7581 | 8.772206 | 0.224163 | -27.9516 | 6.435422 | 2.13E-05 | 7.26E-13 | 623.5458 |
| 115 | ukb-a-60 | finn-b-I9_CORATHER | Coronary atherosclerosis \|\| id:finn-b-I9_CORATHER | \|\| id:ukb-a-60 | Weighted mode | 71 | -9.02908 | 5.357774 | 0.096398 | -19.5303 | 1.47216 | 0.00012 | 3.30E-09 | 4.358639 |
